# Combinations of plant water-stress and neonicotinoids can lead to secondary outbreaks of Banks grass mite (*Oligonychus pratensis* Banks)

**DOI:** 10.1371/journal.pone.0191536

**Published:** 2018-02-28

**Authors:** Alice Ruckert, L. Niel Allen, Ricardo A. Ramirez

**Affiliations:** 1 Department of Biology, Utah State University, Logan, Utah, United States of America; 2 Civil and Environmental Engineering Department, Utah State University, Logan, Utah, United States of America; University of Vienna, AUSTRIA

## Abstract

Spider mites, a cosmopolitan pest of agricultural and landscape plants, thrive under hot and dry conditions, which could become more frequent and extreme due to climate change. Recent work has shown that neonicotinoids, a widely used class of systemic insecticides that have come under scrutiny for non-target effects, can elevate spider mite populations. Both water-stress and neonicotinoids independently alter plant resistance against herbivores. Yet, the interaction between these two factors on spider mites is unclear, particularly for Banks grass mite (*Oligonychus pratensis*; BGM). We conducted a field study to examine the effects of water-stress (optimal irrigation = 100% estimated evapotranspiration (ET) replacement, water stress = 25% of the water provided to optimally irrigated plants) and neonicotinoid seed treatments (control, clothianidin, thiamethoxam) on resident mite populations in corn (*Zea mays*, hybrid KSC7112). Our field study was followed by a manipulative field cage study and a parallel greenhouse study, where we tested the effects of water-stress and neonicotinoids on BGM and plant responses. We found that water-stress and clothianidin consistently increased BGM densities, while thiamethoxam-treated plants only had this effect when plants were mature. Water-stress and BGM herbivory had a greater effect on plant defenses than neonicotinoids alone, and the combination of BGM herbivory with the two abiotic factors increased the concentration of total soluble proteins. These results suggest that spider mite outbreaks by combinations of changes in plant defenses and protein concentration are triggered by water-stress and neonicotinoids, but the severity of the infestations varies depending on the insecticide active ingredient.

## Introduction

The use of neonicotinoid treated seeds has dramatically increased in the United States for the management of insect herbivores in field crops [1], partly due to the systemic and translaminar action of the insecticide, its long residual activity in plants, and reduced exposure of the operator to the insecticide [2]. Recently, neonicotinoid insecticides have come under scrutiny because of their effects on beneficial insects [3–5] and their association with outbreaks of spider mites (Acari: Tetranychidae) [6–11]. Spider mites are important agricultural pests that feed on all major food crops and many ornamental plants. Neonicotinoids are not effective in suppressing spider mites [7] and little effect has been observed on the natural enemies of this pest [12–15]. Two mechanisms that can explain spider mite outbreaks following neonicotinoid applications include the increased production of eggs stimulated by the insecticide [8] and plant-mediated mechanisms thought to involve plant defenses [7]. Szczepaniec et al. [7], for example, found that neonicotinoids alter phytohormone 12-oxo-phytodienoic acid, which is an important precursor of plant defense regulators (e.g., jasmonic acid (JA) and salicylic acid (SA)) that can lead to increased susceptibility of plants to herbivores.

In the western U.S., the effects of neonicotinoids on spider mites are confounded with frequent and severe drought episodes. Under hot and dry conditions, spider mite populations can increase exponentially and reach damaging levels [16–21]. Changes in microhabitat (i.e., increased dust) that favor spider mites can lead to outbreaks during drought conditions and several plant physiological changes (i.e., higher nutrient availability and altered biosynthesis of plant defenses) can also occur that may further contribute to these outbreaks [22–31]. As a consequence, the severity of spider mite outbreaks from the use of neonicotinoids may be dictated by changes in water-stress, and the interaction between these two simultaneously occurring abiotic factors. However, the interactive effect of neonicotinoids and water-stress on the development of spider mite infestations and the physiology of their plant host remain unknown.

Spider mites are a serious pest of corn, especially in the semiarid regions of the western U.S. [32], and their management is becoming increasingly difficult, due to their ability to develop pesticide resistance [33,34]. So far, work has focused mostly on the cosmopolitan twospotted spider mite (*Tetranychus urticae* Koch) (TSSM) and less is known about the Banks grass mite (*Oligonychus pratensis* Banks) (BGM), a grass specialist, and its response to water-stress and neonicotinoid treatments. We tested the combined effect of water-stress, which is prevalent in the Intermountain West and predicted to intensify [35], and neonicotinoids, widely used as a seed treatment in corn, on BGM, which primarily attacks this crop [36]. Field and greenhouse experiments examined (1) how water-stress and the use of neonicotinoid insecticides affect spider mite populations over time, and (2) how these factors influence plant phenotypic and physiological responses in the presence or absence of spider mite herbivory.

## Material and methods

### Field experiment 1: Effect of water-stress and neonicotinoid seed treatments on resident spider mite populations

We conducted a 3×2 factorial experiment (three types of neonicotinoid seed treatments × two irrigation levels) with repeated measures to determine the effects of neonicotinoids and water-stress on resident spider mite populations in corn. This field experiment was conducted in 2013 at the Utah State University Greenville Research Station in Logan, UT, USA.

Experimental units were 2×1.5 m plots arranged in a completely randomized design within varied irrigation levels. Plots receiving the same irrigation treatment were spaced 2 m apart, while plots with different irrigation levels were 4 m apart, necessary to establish two distinct irrigation levels. Each plot was seeded with field corn (*Zea mays* hybrid. KSC7112, relative maturity 112 days; Bayer Crop Science, Raleigh-Durham, NC, USA), treated with the fungicide Evergol Energy (mixture of prothioconazole, penflufen, and metalaxyl; Bayer Crop Science) at a rate of 65.19 ml/100 kg and treated with either clothianidin (‘Poncho’, 0.5 mg a.i./seed; Bayer Crop Science), thiamethoxam (‘Cruiser’, 0.5 mg a.i./seed; Syngenta), or no neonicotinoid (Control). Each plot contained 30 plants divided into 2 rows at a distance of 15 cm within rows and 75 cm between rows. A granular fertilizer (15N:9P:12K; Scotts Osmocote Plus) was applied 3 times (0.5 kg/sq. meter): prior to seeding, when plants had 8-10 fully developed leaves, and when the first corn silk appeared.

Water was provided to plants using drip tape (Toro EAP 5101245-600, 15 mm diameter, 0.10 mm thin black plastic, 30 cm emitters, Q-100: 2.8 × 10^-5^ m^3^s^-1^/30 m at 0.7 bar). All plots were watered at field capacity maintaining 100% replacement of the total water lost by estimated evapotranspiration (ET) during establishment; at three weeks after germination, the two irrigation treatments were initiated. Plants under optimal irrigation were kept at field capacity and received 100% replacement of estimated ET, while water-stressed plants received 25% of the irrigation provided to optimally watered plots, and were approximately kept at wilting point. Potential crop ET was estimated according to Allen et al. [37], where the daily reference evapotranspiration (ETo) is multiplied by the crop coefficient (Kc). The total water provided to the plants was then calculated by subtracting the precipitation recorded within two consecutive irrigation events from the plant irrigation need accumulated in the same time period. With the variation in timing of precipitation events, irrigation timing was adjusted accordingly but generally occurred twice a week. To make sure that the two irrigation levels were maintained over time, visual signs of plant water-stress were monitored (leaf curling) as well as soil moisture, which was measured following the gravimetric sampling method [38]. Data on precipitation were obtained from an onsite weather station (Texas Electronics TR-525I Rain Gauge Tipping Bucket, Dallas, Texas, USA), while data on reference evapotranspiration were provided by Utah Agweather (https://climate.usurf.usu.edu/agweather.php). Each treatment combination was replicated 3 times (*N* = 18).

Plant samples were collected once a week for four weeks, from plant tasseling until the soft dough phase. One fully developed leaf per plant was collected from five destructively sampled plants at each collection date. At each sampling event, we selected the third leaf below the newly developing leaf from the top of each sampled plant, given that spider mites move upward as leaves become older and resource-deficient [39]. Leaf samples were transported to the laboratory on ice and stored in a freezer at -20°C.

Density of resident spider mites (BGM and TSSM) were recorded using a stereomicroscope (Leica S6 D Greenough). Counts were made on both sides of the leaf along the mid-vein by cutting out 36 cm^2^ subsamples (4 per leaf) across the leaf length. The density of mites (number of mites per cm^2^) was calculated by dividing the total number of individuals by the total area examined.

### Field experiment 2: Effect of water-stress and clothianidin seed treatments on BGM and plant responses

Field experiment 2 was conducted to isolate the effects of water-stress and neonicotinoids on BGM specifically, and to evaluate changes in plant responses to each abiotic factor and spider mite herbivory. Field experiment 2 was conducted in 2013 and 2015 at the Greenville Research Station for comparisons across multiple seasons. This experiment was a 2×2×2 factorial experiment (two levels of neonicotinoid treatment × two irrigation levels × mite presence / absence) with repeated measures.

Experimental units were 2×2×2 m lumite cages (530 μ mesh opening) (Lumite, Alto, GA, USA). Each treatment was replicated five times in 2013 (*N* = 40), and three times in 2015 (*N* = 24). Cages were setup in a completely randomized design within each irrigation level. Each cage housed 30 corn plants (*Zea mays* hybrid. KSC7112), distributed into two rows at a distance of 15 cm within rows and 75 cm between rows. Cages excluded other herbivores and predators from the experiment. Plots receiving the same irrigation treatment were spaced 2 m apart, while cages with different irrigation levels were 4 m apart, necessary to establish two distinct irrigation levels.

In this experiment, half of the seeds were treated with clothianidin (‘Poncho’, 0.5 mg a.i./seed), while the other half were left untreated. Irrigation treatments were setup as described in field experiment 1 using drip tape, and fertilizer was applied to all cages as previously discussed.

Four weeks after germination (vegetative stage V5), ten adult BGM females were transferred to each plant with a fine paintbrush. BGM colonies were established from individuals collected in 2012 from commercial field corn and reared on untreated corn plants under laboratory conditions (16:8h light:dark photoperiod and 28±2°C). After one week of BGM establishment, the second and third fully developed leaves below the newly developing leaf were collected from each of four destructively sampled plants. Leaf samples were obtained each week for 5 weeks from four unique plants until tasseling. Leaves were stored in a freezer at -20°C so that BGM could be recorded and plant defense compounds evaluated at a later date (see Plant Defense Bioassay section). BGM were counted on both sides of the leaf along the mid-vein by taking 0.785 cm^2^ subsamples evenly distributed across the length of the leaf. Subsamples were 1.5 cm apart from each other. The density of BGM (number of BGM per cm^2^) was calculated by dividing the total number of individuals by the total area examined.

### Field experiment 3: A Comparison of clothianidin and thiamethoxam effects on BGM and plant responses exposed to water-stress

Field experiment 3 was conducted to determine whether effects of neonicotinoids on BGM with water-stressed plants were consistent across different neonicotinoid active ingredients. Here, experiment 3 was a 3×2×2 factorial experiment (three levels of neonicotinoid treatment × two irrigation levels × mite presence / absence) with repeated measures. This experiment utilized all treatments described for field experiment 2 in 2015, adding a neonicotinoid treatment, thiamethoxam (‘Cruiser’, 0.5 mg a.i./seed), which provided a way to make comparisons between neonicotinoid active ingredients. Each treatment was replicated three times (N = 36).

Similar to field experiment 2, the experimental units were 2×2×2 m lumite cages containing 30 plants and the experimental setup in all respects (i.e., treatment of seed, irrigation, BMG establishment, and leaf sampling) were the same.

For experiment 3, stem height, leaf number and area, leaf temperature, and yield (cob dry weight) were also measured, as well as the concentration of neonicotinoids in leaf tissue. Plant height was measured from the soil surface to the last fully developed leaf on the stalk, and all fully developed leaves were counted. Leaf area was calculated using ImageJ Software (version 1.41, National Institutes of Health, Bethesda, Maryland, USA), while leaf temperature, which is used to detect water-stress conditions in plants [40], was measured using an infrared thermometer (Cen Tech, model number 69465, Temecula, CA, USA) following Stiefel [41]. Yield was determined by measuring the dry weight of the cob after desiccation in a drying oven at 60°C for 14 days. Finally, the concentration of clothianidin and thiamethoxam were quantified in leaf tissues not subjected to BGM herbivory using ELISA (Enzyme Linked Immunosorbent Assay, SmartAssay Series Test Kit, HORIBA, Ltd, Kyoto, Japan).

### Greenhouse experiment 1: Effect of water-stress and neonicotinoid seed treatments on BGM and plant responses

Greenhouse experiment 1, which was conducted at the Utah State University Research Greenhouse in Logan, UT, mirrored field experiment 3 and evaluated the combined effect of water-stress and neonicotinoids on BGM populations and plant defenses.

Greenhouse experiment 1 was setup as a 3×2×2 factorial experiment (three types of neonicotinoid treatment × two irrigation levels × mite presence / absence) and each treatment was replicated three times (*N* = 36).

Experimental units were represented by plants grown within 5-liter capacity pots (22.5 cm top diameter, 16.5 cm base diameter, 17.8 cm depth), distributed in a completely randomized design. Each pot received a single seed (*Zea mays* hybrid. KSC7112) grown within Sunshine soil mix #3 as substrate and under 14:10 h light:dark photoperiod. Fertilization was applied once at seeding with Scotts Osmocote time release fertilizer (14:14:14, N-P-K).

Similar to the field experiments, plants receiving optimal irrigation were kept at field capacity, which is equal to a volumetric water content (VWC) of 15% for sandy-loam soils, while plants under water-stress conditions were kept at 5%VWC, where the wilting point is reached in the same soil type [42]. VWC was measured with a soil moisture sensor probe (FieldScout TDR 100, Spectrum Technologies, Inc) and the two irrigation treatments were initiated three weeks after germination.

Ten adult BGM females were transferred to each of three leaves per plant four weeks after germination. A plastic sleeve (ClearBags 33×10 cm micro perforated bags, model MPF1324) was used to enclose each leaf. After three weeks, leaves were collected and BGMs were counted on both sides of the entire leaf. BGM density was then estimated to the leaf level. Plant height, leaf number, leaf area, neonicotinoid concentration in leaf tissue, and plant defense proteins were recorded. Leaf temperature was also recorded, and measurements occurred just before each irrigation treatment within the plastic sleeve cover and on the adjacent leaf not enclosed in the sleeve for comparison.

### Greenhouse experiment 2: Effect of water-stress and neonicotinoid seed treatments on BGM fecundity

Greenhouse experiment 2 was conducted to evaluate the effect of water-stress and neonicotinoids on the fecundity of BGM. Greenhouse experiment 2 was setup as a 3×2 factorial experiment (three types of neonicotinoid treatment × two irrigation levels) with each treatment replicated four times (*N* = 24). Here, the experimental units (5-liter capacity pots with a single plant) and setup with regard to seed treatment, irrigation, and randomization in the greenhouse were the same as described for greenhouse experiment 1.

For greenhouse experiment 2, a single adult unmated female BGM was transferred to each of five leaves per plant, six weeks after germination. These leaves were enclosed with a plastic sleeve, collected ten days later, and then the total number of eggs and juveniles was recorded. The collection timing prevented the start of a second BGM generation, following Mondal and Ara [43].

### Plant defense bioassays

All collected leaves were analyzed for levels of total soluble proteins, as well as plant protein defense compounds including peroxidase (POD), polyphenol oxidase (PPO), trypsin protease inhibitor (TI), and chitinase (CHI). POD and CHI are directly related to the phytohormone pathways of JA, while PPO and TI are regulated by SA [44]. Proteins were extracted by macerating 15 mg of leaf material in 0.25 mL of 0.05M sodium phosphate buffer (pH 7.0) and analyzed following methods in Bradford [45]. POD, PPO, and CHI were measured using a microplate reader (Biotek EPOCH, Winooski, VT, USA), following methods in Moran and Cipollini [46] and dal Soglio et al. [47]. TI was measured by examining the diffusion of protein extracts through an agar substrate and analyzed following methods in Cipollini and Bergelson [48].

## Statistical analysis

For field experiment 1, a two-way ANOVA with repeated measures that included WATER (+,-) and PESTICIDE (control, clothianidin, thiamethoxam) as factors, was used to test differences in BGM density across the treatments.

Field experiment 2 was analyzed within a 2×2×2 factorial design (two levels of neonicotinoid treatment × two irrigation levels × two trials) with repeated measures across two seasons (2013 and 2015) to evaluate differences between clothianidin and control plants (non-neonicotinoid treated) on BGM density. For field experiment 2, plant protein data were analyzed using a three-way ANOVA with repeated measures which included WATER (+,-), PESTICIDE (control, clothianidin) and HERBIVORY (+,-) as factors.

Field experiment 3, instead, was analyzed within a 3×2 factorial design (three types of neonicotinoid treatment × two irrigation levels) with repeated measures, to evaluate differences between clothianidin and thiamethoxam on BGM density. For field experiment 3, a three-way ANOVA with repeated measures that included WATER (+,-), PESTICIDE (control, clothianidin and thiamethoxam) and HERBIVORY (+,-) as factors, was used to test differences of plant height, leaf area and number, leaf temperature, and yield across treatments. Similar to field experiment 2, plant protein data for field experiment 3 were analyzed using the same statistical method, which accounted for an additional neonicotinoid treatment, thiamethoxam. A one-way ANOVA with repeated measures, that included WATER (+,-) as a factor, was applied to test for differences in the neonicotinoid concentration in field experiment 3.

In greenhouse experiment 1, differences in BGM density, plant height, leaf area and number, leaf temperature, and plant proteins across the treatments were tested with a three-way ANOVA, that included WATER (+,-), PESTICIDE (control, clothianidin, thiamethoxam) and HERBIVORY (+,-) as factors. A two-sample t-test with unequal variances was used to test for differences in the neonicotinoid concentration of plant tissues that were not subjected to spider mite herbivory, but were kept under optimal water conditions or water-stress.

In greenhouse experiment 2, a two-way ANOVA with repeated measures that included WATER (+,-) and PESTICIDE (control, clothianidin, thiamethoxam) as factors, was used to test differences in egg abundance across the treatments.

To meet model assumptions for all experiments, data were log transformed and analyzed using SAS (version 9.3; SAS Institute Inc., Cary, NC, USA). Significant interaction terms were examined with Holm’s step-down Bonferroni.

## Results

### Field experiment 1: Effect of water-stress and neonicotinoid seed treatments on resident spider mite populations

**Fig 1:**
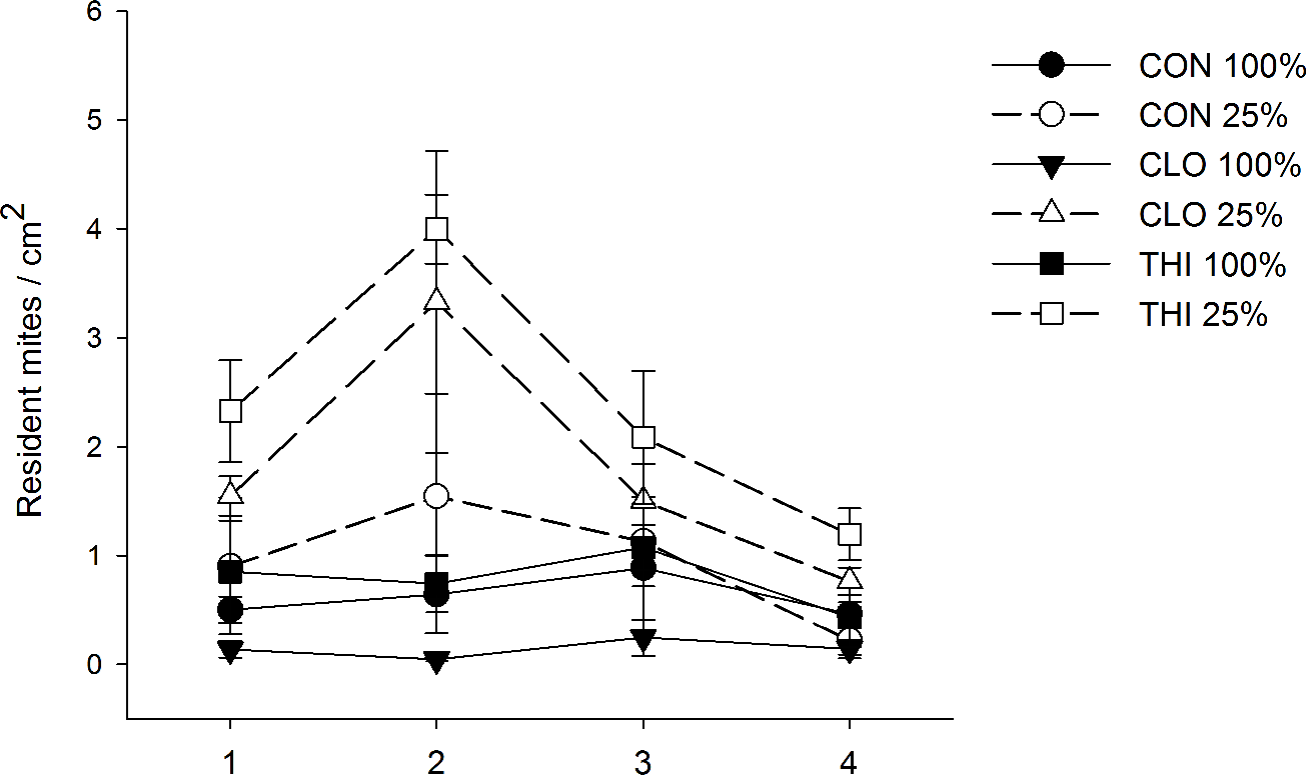
Effect of water-stress and neonicotinoids on density (mean ± 1 S.E.) of resident mites. Circles represent control (CON - no insecticide treatment); triangles and squares represent plants treated with clothianidin (CLO) and thiamethoxam (THI), respectively. Optimal irrigation (100% estimated ET replacement) is represented by solid lines and symbols, and water-stress (25% of the total water provided to optimally irrigated plants) is represented by dashed lines, open symbols.

Nearly 96% of the resident spider mite population was represented by BGM, while 4% was identified as TSSM. Water-stressed plants treated with either clothianidin or thiamethoxam had higher resident spider mite density than all other treatments (Field Experiment 1- WATER×PESTICIDE: *F*_*2*, *44*_ = 8.65, *P*< 0.001; Holm’s step down Bonferroni comparisons P< 0.03) (Fig 1).

### Field experiment 2: Effect of water-stress and clothianidin seed treatments on BGM and plant responses

**Fig 2:**
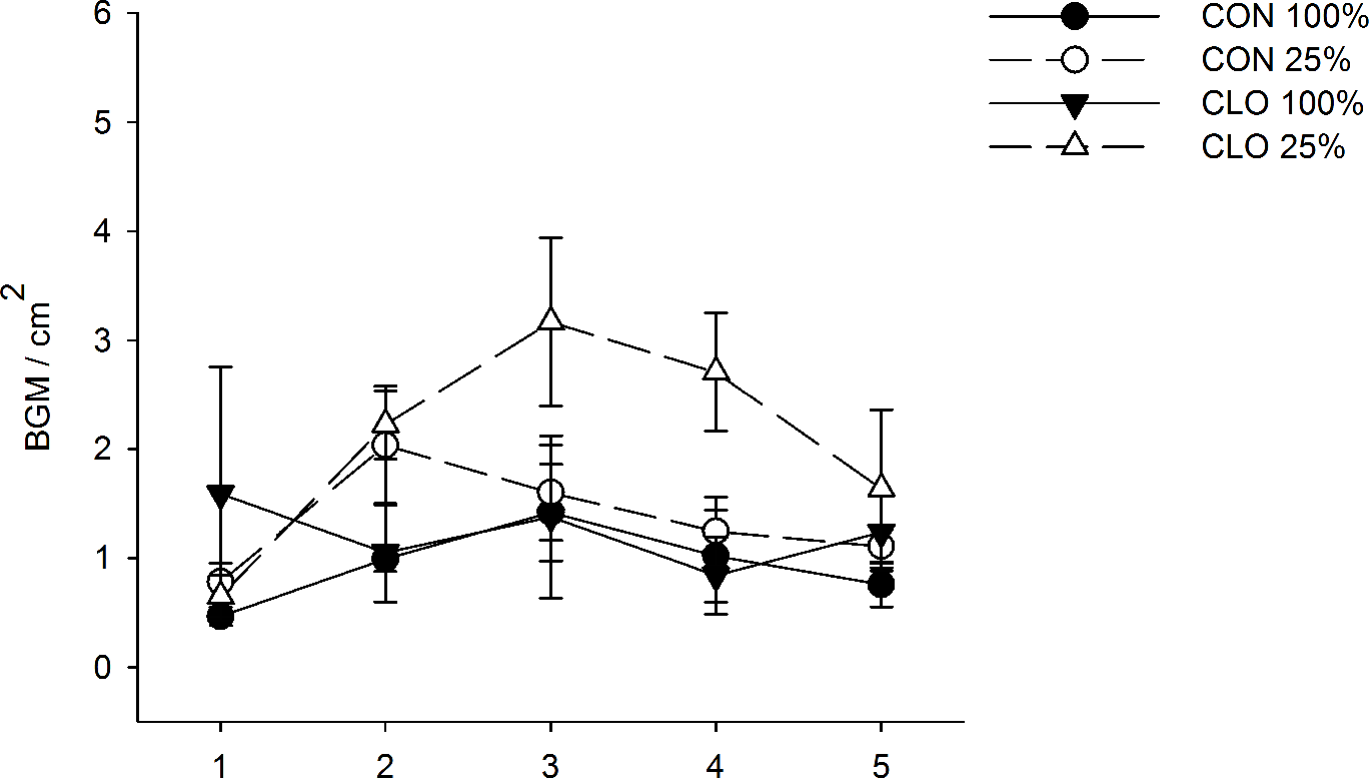
Effect of water-stress and neonicotinoids on density (mean ± 1 S.E.) of BGM. Circles represent control (CON - no insecticide treatment); triangles represent plants treated with clothianidin (CLO). Optimal irrigation (100% estimated ET replacement) is represented by solid lines and symbols, and water-stress (25% of the total water provided to optimally irrigated plants) is represented by dashed lines, open symbols.

BGM densities (2013 and 2015) increased throughout the season, a result that was apparently driven when BGM were exposed to water-stressed plants treated with clothianidin (WATER×PESTICIDE×TIME: *F*_*4*, *101*_ = 2.74, *P* = 0.03) (Fig 2).

#### Plant proteins

**Fig 3:**
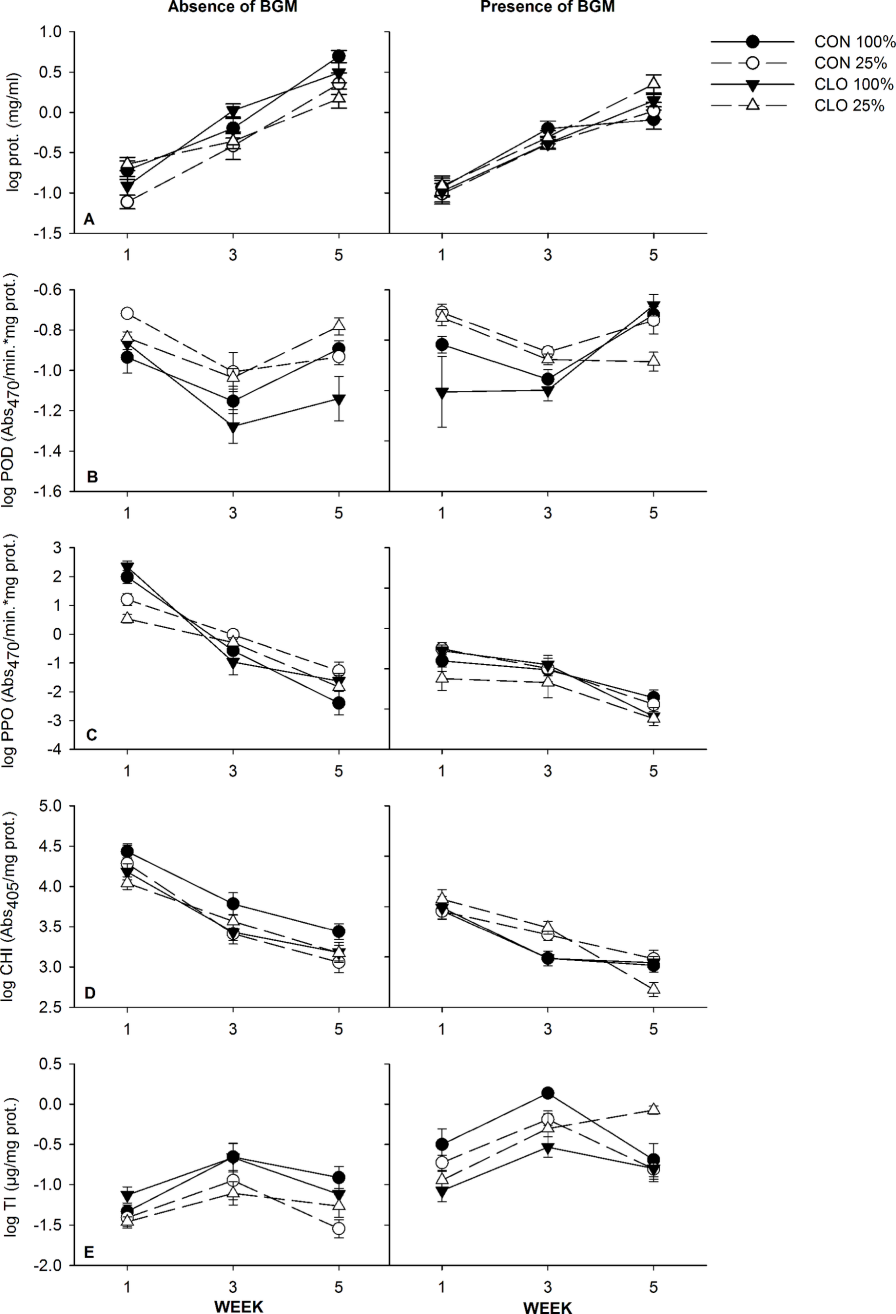
Effects of water-stress, neonicotinoids, and BGM herbivory on plant defense proteins (Field experiment 2) (Mean ± 1 S.E.). POD, peroxidase; PPO, polyphenol oxidase; CHI, chitinase; TI, trypsin inhibitor. Circles represent control (CON - no insecticide treatment); triangles represent plants treated with clothianidin (CLO). Optimal irrigation (100% estimated ET replacement) is represented by solid lines and symbols, and water-stress (25% of water provided to optimally irrigated plants) is represented by dashed lines, open symbols.

In field experiment 2, we found that the total protein concentration appeared to increase more rapidly throughout the season in plants treated with clothianidin than in non-treated control plants when BGM was present (PESTICIDE×HERBIVORY×TIME: *F*_*2*, *93*_ = 3.79, *P* = 0.03) (Fig 3A, S1 Table). In the absence of BGM, the total protein concentration appeared to increase at a similar rate in all plant treatments throughout the season.

Plants provided with optimal irrigation appear to have lower concentrations of POD than water-stressed plants throughout the season when BGM was absent. Instead the presence of BGM appear to increase POD concentrations, however this result was observed in well-watered plants only (WATER×HERBIVORY×TIME: *F*_*2*, *90*_ = 2.49, *P* = 0.09) (Fig 3B, S2 Table).

PPO concentrations appeared to decrease over time and the magnitude of this change was less for plants with BGM, as BGM herbivory appeared to decrease PPO within the first week, compared to plants without BGM (HERBIVORY×TIME: *F*_*2*,*93*_ = 36.04, *P<* 0.0001; Holm’s step down Bonferroni *P*< 0.0001) (Fig 3C, S3 Table).

Similar to observations for PPO, CHI concentrations appeared to decrease over time and the magnitude of this change was less for plants with BGM, as the presence of BGM appeared to reduce CHI within the first week (HERBIVORY×TIME: *F*_*2*, *62*_ = 5.71, *P<* 0.01; Holm’s step down Bonferroni *P*< 0.05) (Fig 3D, S4 Table).

Water-stress appeared to reduce TI concentrations when BGM were absent, while in the presence of herbivory concentrations of TI increased, especially in water-stressed plants (WATER×HERBIVORY: *F*_*1*,*93*_ = 3.30, *P* = 0.07) (Fig 3E, S5 Table).

### Field experiment 3: A Comparison of clothianidin and thiamethoxam effects on BGM and plant responses exposed to water-stress

**Fig 4:**
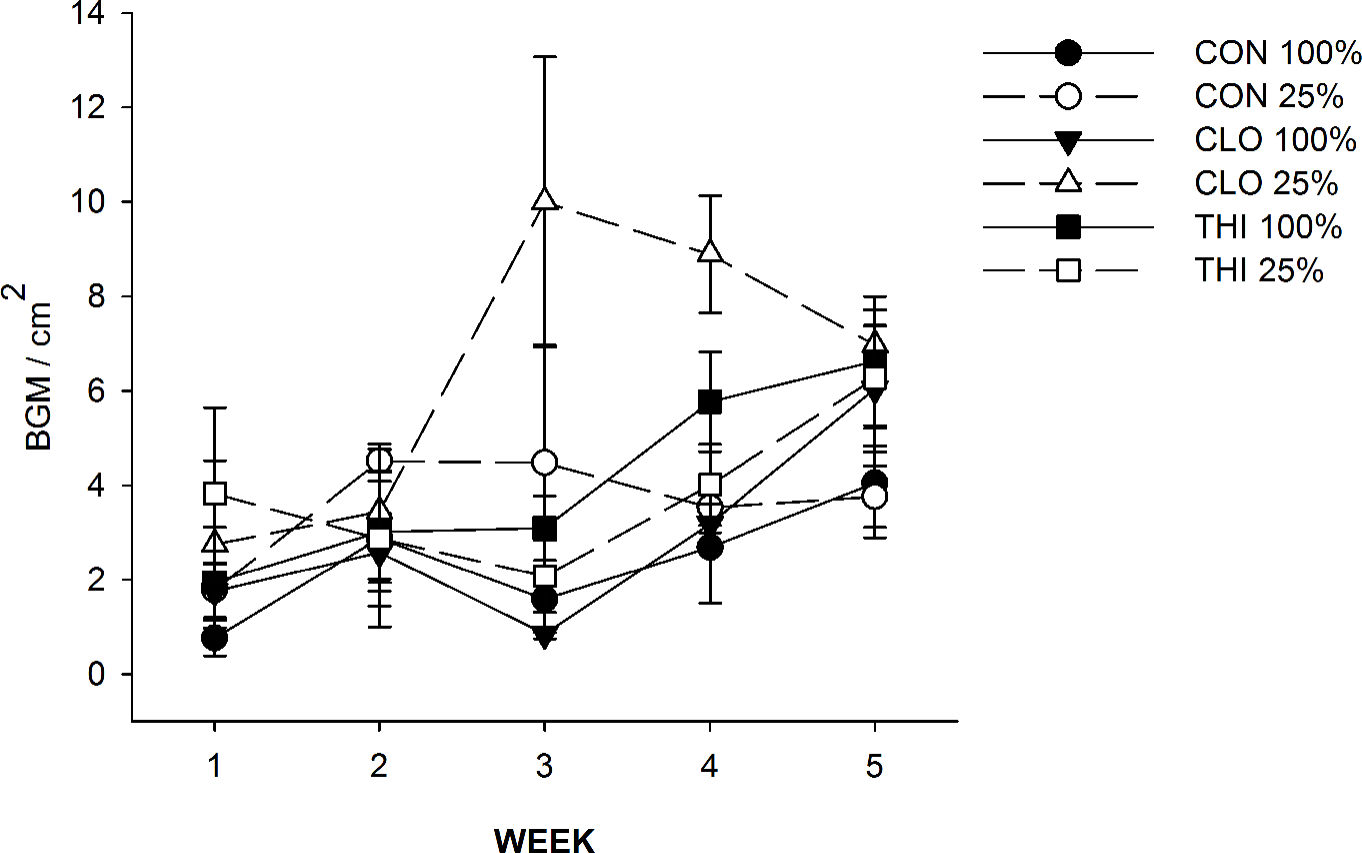
Effect of water-stress and neonicotinoids on density (mean ± 1 S.E.) of BGM. Circles represent control (CON - no insecticide treatment); triangles and squares represent plants treated with clothianidin (CLO) and thiamethoxam (THI), respectively. Optimal irrigation (100% estimated ET replacement) is represented by solid lines and symbols, and water-stress (25% of the total water provided to optimally irrigated plants) is represented by dashed lines, open symbols.

Upon further evaluation of each neonicotinoid seed treatment (clothianidin vs. thiamethoxam), BGM densities did not increase when exposed to thiamethoxam as they did with clothianidin and were similar to the control plants (WATER×PESTICIDE×TIME: *F*_*8*, *48*_ = 2.11, *P* = 0.05) (Fig 4).

#### Phenotypic plant responses

Water-stressed plants had reduced plant height (WATER×DATE: *F*_*1*, *24*_ = 104.74 *P<* 0.0001), leaf number (WATER: *F*_*1*, *24*_ = 104.74 *P<* 0.0001), leaf area (WATER: *F*_*1*, *120*_ = 19.54, *P<* 0.0001), and yield (WATER: *F*_*1*, *24*_ = 171.14, *P<* 0.0001). Specifically, yield was decreased by nearly 54%.

Feeding by BGM also decreased the leaf area (HERBIVORY: *F*_*1*, *120*_ = 11.64, *P<* 0.001) and yield (HERBIVORY: *F*_*1*, *24*_ = 18.75, *P<* 0.001). Specifically, BGM reduced yields by nearly 20%. Moreover, the combination of BGM and thiamethoxam had significantly smaller cobs than thiamethoxam-treated plants without mites (Holm’s step down Bonferroni *P*< 0.001).

Interestingly, water-stress and presence of BGM significantly increased leaf temperature (WATER: *F*_*1*, *120*_ = 18.43 *P<* 0.0001, HERBIVORY: *F*_*1*, *120*_ = 9.68 *P<* 0.01). Water-stressed plants had elevated leaf temperatures of 0.73 ± 0.2°C, compared to plants provided with optimal irrigation. BGM also increased leaf temperature by 0.5 ± 0.3°C, compared to plants without BGM.

#### Plant proteins

**Fig 5:**
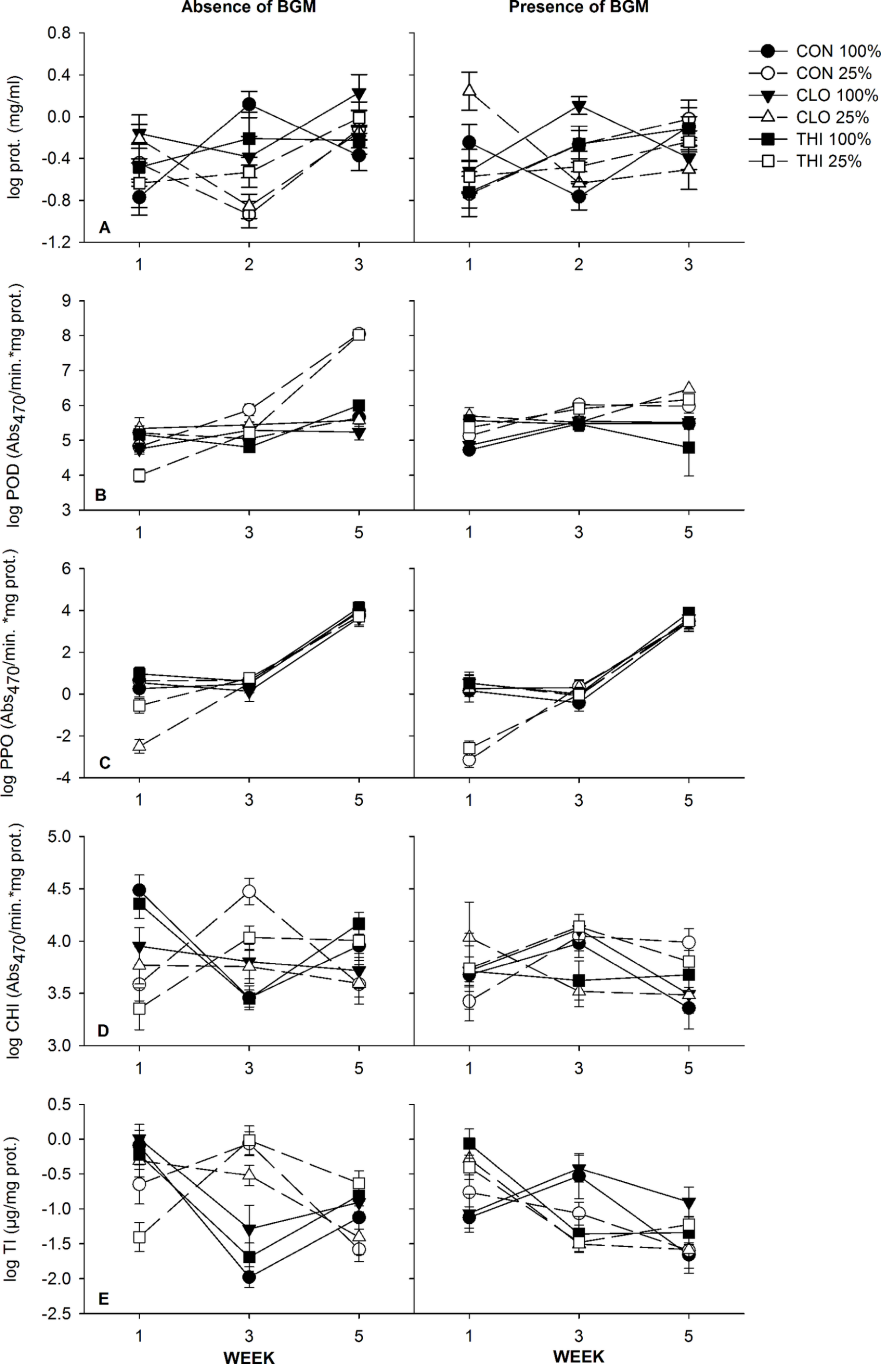
Effects of water-stress, neonicotinoids, and BGM herbivory on plant defense proteins (Field experiment 3) (mean ± 1 S.E.). POD, peroxidase; PPO, polyphenol oxidase; CHI, chitinase; TI, trypsin inhibitor). Circles represent control (CON - no insecticide treatment); triangles and squares represent plants treated with clothianidin (CLO) and thiamethoxam (THI) respectively. Optimal irrigation (100% estimated ET replacement) is represented by solid lines and symbols, and water-stress (25% of water provided to optimally irrigated plants) is represented by dashed lines, open symbols.

In field experiment 3, plants provided with optimal irrigation appeared to have a higher protein concentration when BGM were absent, however the effect was lost over time (WATER×HERBIVORY×TIME: *F*_*2*, *72*_ = 4.66, *P* = 0.01) (Fig 5A, S6 Table). In the presence of BGM, well-watered plants and water-stressed plants appeared to have instead a similar protein concentration throughout the season.

POD concentrations were the same throughout the season for all plants receiving optimal irrigation. However, when plants were subjected to water-stress, POD concentrations appeared to increase in control and in thiamethoxam treatments, while POD concentrations appeared to remain the same in plants treated with clothianidin (WATER× PESTICIDE×TIME: *F*_*4*,*68*_ = 5.06, *P<* 0.01) (Fig 5B, S7 Table).

PPO concentrations were affected by all factors, hence interpretation was complex and could not be described further (WATER×PESTICIDE×HERBIVORY: *F*_*2*,*24*_ = 8.86, *P<* 0.01) (Fig 5C, S8 Table). Overall, however, PPO concentrations appeared to increase over time (TIME: *F*_*2*,*72*_ = 267.29, *P<* 0.0001).

The absence of BGM appeared to increase CHI concentrations in plants receiving optimal irrigation more than water-stressed plants, but the effect was lost over time as CHI concentrations decreased and then stabilized. In the presence of BGM, no difference between the two irrigation levels was observed throughout the season (WATER×HERBIVORY×TIME: *F*_*2*, *72*_ = 6.15, *P<* 0.01) (Fig 5D, S9 Table). In general, we found that control plants and thiamethoxam-treated plants appeared to have similar CHI concentrations regardless of mite herbivory, compared to clothianidin-treated plants.

Similar to field experiment 2, we found that water-stress alone initially decreased TI concentrations compared to plants provided with optimal irrigation, but the effect of water-stress was short lived as it dissipated over time (WATER×TIME: *F*_*1*,*71*_ = 3.48, *P*< 0.05) (S10 Table). Contrary to field experiment 2, the interactive effect of BGM herbivory and water-stress was not detected (Fig 5E).

#### Neonicotinoid concentration

There was no difference in the concentration of clothianidin and thiamethoxam in leaf tissue of plants not subjected to BGM between the two irrigation levels (WATER: *F*_*1*, *4*_ = 1.85 *P* = 0.25 and *F*_*1*,*4*_ = 0.01 *P* = 0.93 respectively). Clothianidin strongly decreased throughout the season from an average of 30±3.84 ppb to approximately 15±5.10 ppb (TIME: *F*_*4*, *15*_ = 2.99 *P* = 0.05), while thiamethoxam did not change over time and maintained an average of 16 ± 1.06 ppb (TIME: *F*_*4*, *16*_ = 0.99 *P* = 0.44).

### Greenhouse experiment 1: Effect of water-stress and neonicotinoid seed treatments on BGM

**Fig 6:**
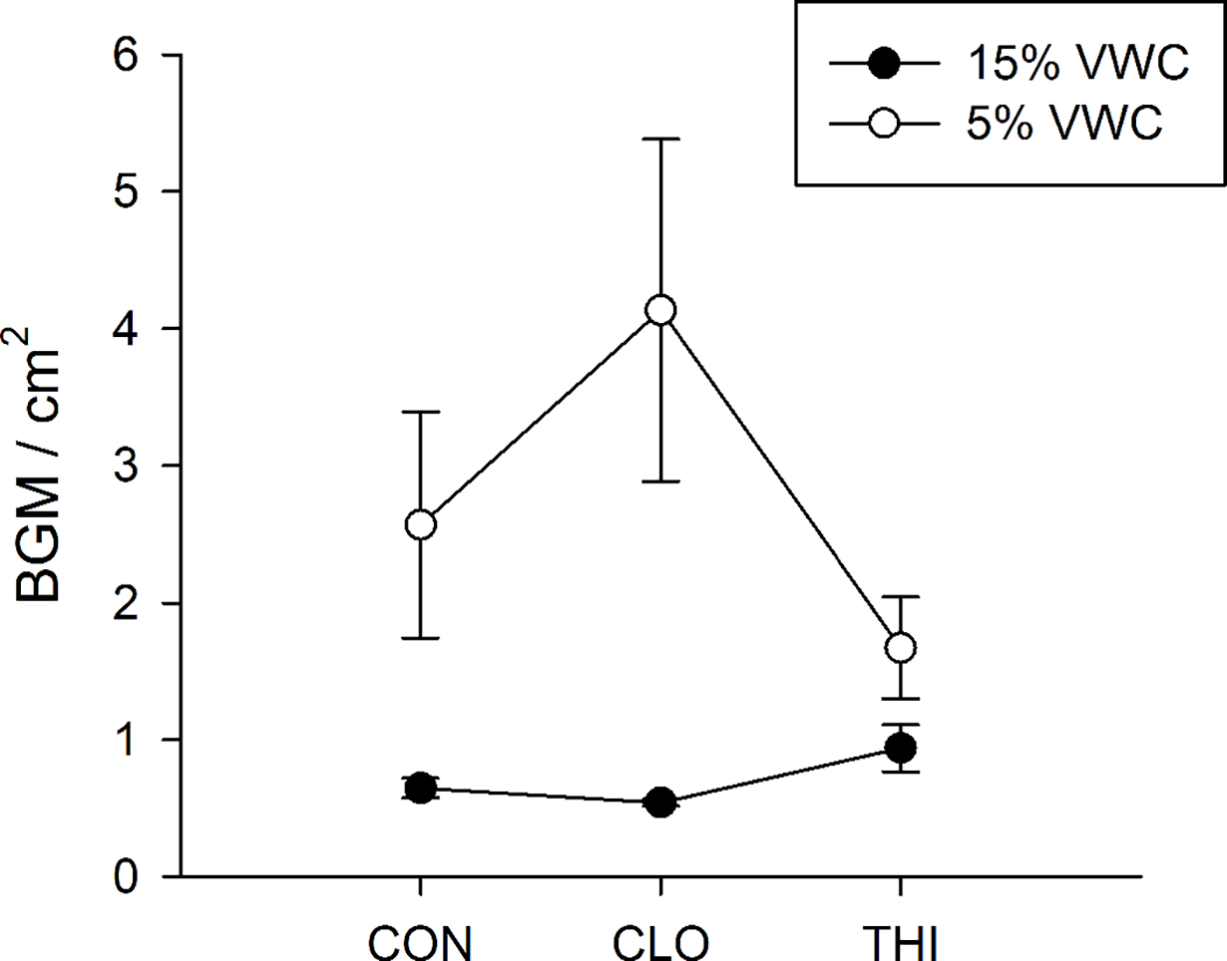
Effect of water-stress and neonicotinoids on the density of BGM (mean ±1 S.E.). Soil volumetric water content (VWC) is expressed in %. Solid symbols represent plants provided with optimal irrigation (15% VWC); open symbols represent water-stressed plants (5% VWC); CON = control; CLO = clothianidin; THI = thiamethoxam. *Lowercase letters* represent step-down Bonferroni significant differences (*P*< 0.05) within a treatment.

In greenhouse experiment 1, water-stressed plants had more BGMs than well-watered plants, and the combination of water-stress and clothianidin increased BGM populations but this was not significantly different from control or thiamethoxam (WATER×PESTICIDE: *F*_*2*, *12*_ = 4.21, *P* = 0.04; Holm’s step down Bonferroni comparisons *P*< 0.02) (Fig 6). Interestingly, no difference was observed between the two irrigation levels in thiamethoxam-treated plants.

#### Phenotypic plant responses

Water-stressed plants had reduced plant height (WATER: *F*_*1*_ = 654.92 *P<* 0.0001), leaf number (greenhouse: WATER: *F*_*1*_ = 561.72 *P<* 0.0001), and leaf area (WATER: *F*_*1*_ = 81.96, *P<* 0.0001). Feeding by BGM also decreased the leaf number (HERBIVORY: *F*_*1*_ = 4.96 *P* = 0.04) and leaf area (HERBIVORY: *F*_*1*_ = 15.70, *P<* 0.001)

Interestingly, water-stress and presence of BGM significantly increased leaf temperature (WATER: *F*_*1*, *69*_ = 24.36 *P<* 0.0001 and HERBIVORY: *F*_*1*, *69*_ = 11.85 *P<* 0.01). Water-stressed plants had elevated leaf temperatures of 1.05 ± 0.2°C, compared to plants provided with optimal irrigation. BGM also increased leaf temperature by 0.73 ± 0.2°C, compared to plants without BGM.

#### Plant proteins

**Fig 7:**
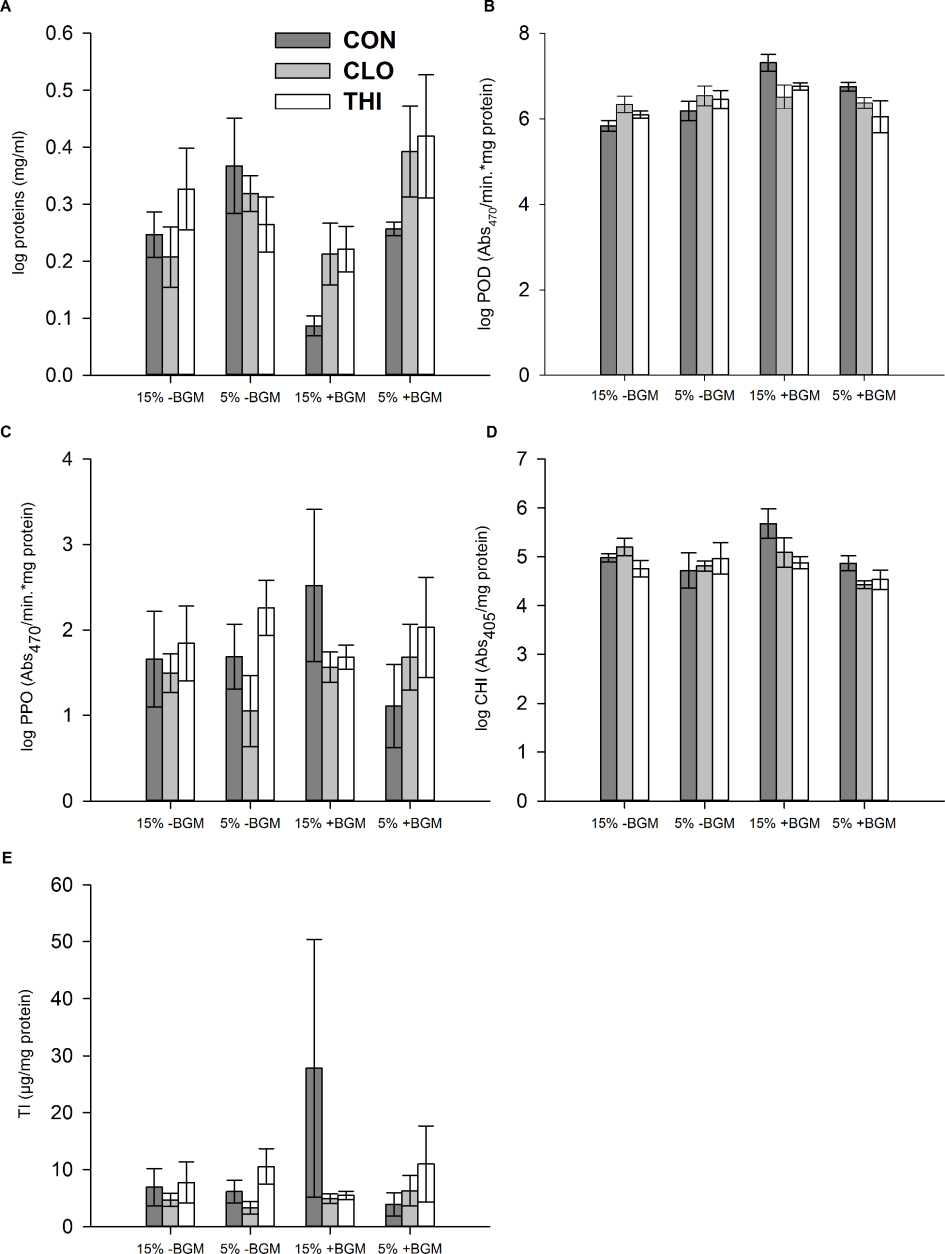
Effects of water-stress, neonicotinoids, and BGM herbivory on plant defense proteins (greenhouse experiment 1) (mean ± 1 S.E.). Soil volumetric water content (VWC) expressed in %, where H- represents plants provided with optimal irrigation (15% VWC) and without BGM; L- represents water-stressed plants (5% VWC) without BGM; H+ represents plants provided with optimal irrigation (15% VWC) and with BGM; L+ represents water-stressed plants (5% VWC) with BGM; dark grey bars represent control (CON - no insecticide treatment), light grey bars represent plants treated with clothianidin (CLO); white bar represent plants treated with thiamethoxam (THI).

Similar to field experiment 2, plants grown in the greenhouse that were treated with either clothianidin or thiamethoxam had a higher concentration of proteins than control plants when BGM were present, while no difference in the total protein concentration was observed across the treatments when BGM was absent (PESTICIDE×HERBIVORY: *F*_*1*, *24*_ = 4.29, *P* = 0.03; Holm’s step down Bonferroni *P*< 0.05) (Fig 7A). Moreover, water-stressed plants had more proteins than plants provided with optimal irrigation in the presence of BGM, while no difference in the total protein concentration was found between the two irrigation levels when BGM was absent (WATER×HERBIVORY: *F*_*1*, *24*_ = 5.40, *P* = 0.03; Holm’s step down Bonferroni *P*< 0.05) (Fig 7A).

Similar to field experiment 2, BGM herbivory in the greenhouse increased POD concentration, particularly on well-watered plants, compared to plants provided with optimal irrigation and no herbivore pressure (WATER×HERBIVORY: *F*_*1*, *24*_ = 10.88, *P<* 0.01; Holm’s step down Bonferroni *P*< 0.01) (Fig 7B). Changes in PPO concentrations were not evident in the greenhouse (Fig 7C).

Similar to field experiment 3, water-stress significantly decreased CHI concentrations compared to plants provided with optimal irrigation (WATER: *F*_*1*, *24*_ = 8.87, *P<* 0.01) (Fig 7D).

TI concentrations were affected by all factors, hence interpretation of the data was complex and could not be further described (WATER×PESTICIDE×HERBIVORY: *F*_*2*, *24*_ = 3.48, *P<* 0.05) (Fig 7E).

#### Neonicotinoid concentration

There was no difference in the concentration of clothianidin and thiamethoxam in leaf tissue of plants not subjected to BGM between the two irrigation levels (WATER: *T*_*4*_ = 3.18 *P* = 0.66 and *T*_*4*_ = 4.30 *P* = 0.25 respectively). The concentration of clothianidin was 4.90 ± 0.36 ppb, while the concetration of thiamethoxam was 3.52 ± 0.43 ppb.

### Greenhouse experiment 2: Effect of water-stress and neonicotinoid seed treatments on BGM fecundity

**Fig 8:**
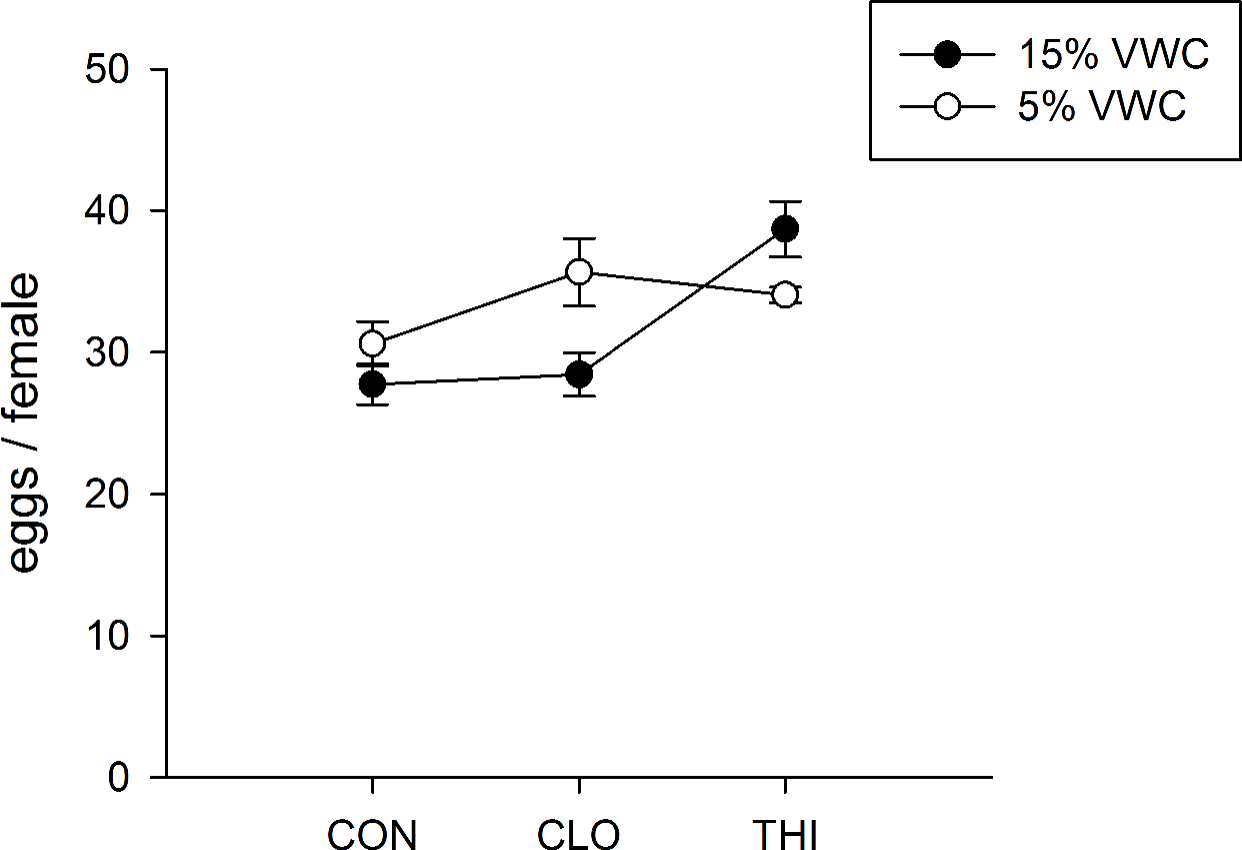
Effect of water-stress and neonicotinoids on the fecundity of BGM (mean ±1 S.E.). Soil volumetric water content (VWC) is expressed in %. Solid symbols represent plants provided with optimal irrigation (15% VWC); open symbols represent water-stressed plants (5% VWC); CON = control; CLO = clothianidin; THI = thiamethoxam. *Lowercase letters* represent step-down Bonferroni significant differences (*P*< 0.05) within a treatment.

BGM deposited more eggs on water-stressed plants, particularly when they were treated with clothianidin (WATER×PESTICIDE: *F*_*2*, *18*_ = 6.03, *P<* 0.01) (Fig 8). We recorded approximately 22% more eggs on these plants than on plants provided optimal irrigation (Holm’s step down Bonferroni *P* = 0.04). Interestingly, thiamethoxam significantly increased the number of eggs on optimally irrigated plants (Holm’s step down Bonferroni *P* = 0.05). No difference in egg abundance was detected between the two irrigation levels on control and thiamethoxam-treated plants (Holm’s step down Bonferroni *P* = 0.24).

## Discussion

Spider mite outbreaks have been regularly observed on plants exposed to hot and dry conditions [6–21], or as a consequence of applying neonicotinoid insecticide [6–11]. We found that the combination of water-stress and neonicotinoid use exacerbated spider mite density on corn. However, BGM outbreaks were not stimulated by the use of neonicotinoids alone, compared to what has been previously observed for TSSM feeding on well-watered cotton and corn plants treated with clothianidin and thiamethoxam [7]. BGM outbreaks occurred only when neonicotinoid-treated plants were water-stressed. The neonicotinoid clothianidin, in particular, had the strongest and most consistent effect on spider mites. This outcome does not appear to be a result of increased neonicotinoid concentration in leaf tissues of water-stressed plants, since non-stressed plants (optimal irrigation) had equal neonicotinoid concentrations in leaves. It is important to note that water-stress and neonicotinoid effects on spider mite outbreaks was not universal, because thiamethoxam-treated plants did not always result in spider mite outbreaks. Specifically, we observed that only the resident spider mite population increased when combining water-stress and thiamethoxam. Nauen et al. [49] found that thiamethoxam metabolizes to clothianidin over time and reaches toxicologically relevant concentrations, which can lead to hormoligosis. In field experiment 1, leaf samples were collected later in the season (between tasseling and the soft dough phase), while they were collected much earlier in all other experiments (from the beginning of the vegetative growth stage until tasseling). One possibility is that thiamethoxam-treated plants in field experiment 1 accumulated the drought interactive clothianidin compound in more mature plants, leading to increased spider mite densities, but this was not specifically tested. Similar to our study, Szczepaniec et al. [7] found that thiamethoxam seed treatments did not elevate *Tetranychus cinnabarinus* (Boisduval) infesting cotton plants. However, thiamethoxam-treated plants appeared to have more mites than control at the end of the sampling period, but this result was not further discussed.

Overall, water-stress was a major factor elevating spider mite densities. We found that corn subjected to drought conditions generally had a higher leaf temperature than plants provided with optimal irrigation. Greater spider mite infestations commonly occur under high temperatures, where both BGM and TSSM lay more eggs and the overall generation time period is shorter [50,51]. In a study conducted under dryland conditions, Stiefel [41] demonstrated that drought-susceptible sorghum lines had a faster development of BGM infestations, due to greater leaf temperatures, than drought-resistant lines. It is possible that, by having a higher leaf temperature, water-stressed plants promoted BGM development. In our experiments, water-stress also altered the concentration of plant defense proteins. Similar to previous studies, water-stress increased the concentrations of POD and TI, as seen in corn and amaranth (*Amaranthus hypochondriacus*) plants respectively [23,24]. This was not surprising as, in some instances, plants can produce more herbivore-associated defenses when water-stressed [52], instead of generally decreasing the biosynthesis of secondary compounds [22]. Unlike what was observed in sesame (*Sesamum indicum*), Madagascar periwinkle (*Catharanthus roseus*), and tomato (*Solanum lycopersicum*), water-stress did not increase PPO and CHI concentrations, [25–27].

Clothianidin and thiamethoxam did not increase BGM densities with optimal irrigation, which is contradictory to Szczepaniec et al. [7], who found a stimulating effect of these two active ingredients on spider mite development in optimally irrigated plants. Szczepaniec et al. [7], however, focused on *T*. *cinnabarinus* and TSSM, where species specific effects may be a possible factor for not observing a similar effect of neonicotinoids when combined with optimal irrigation.

We did not find a clear correlation between spider mite outbreaks and the alteration of plant defense compounds due to neonicotinoids. Clothianidin and thiamethoxam are known to affect plant defense phytohormones by enhancing SA and inhibiting the synthesis of JA from cross-talk [53]. We did not find a tradeoff in PPO and TI, which are regulated by SA, and POD and CHI, which are in turn regulated by JA [44], as cross-talk may predict. Instead, our results are similar to those of Szczepaniec et al. [7], who found that clothianidin did not alter the transcription of CHI (JA-related plant defense) and TI (SA related) in corn. Interestingly, thiamethoxam-treated plants were more similar to control plants than clothianidin-treated plants in terms of plant defense concentrations. For example, the concentration of POD and CHI, which are known to be directly related to spider mite feeding [54], increased over time in water-stressed control and thiamethoxam-treated plants, while their concentrations stayed constant in clothianidin-treated plants under the same water regime. This outcome may explain the increased BGM density on water-stressed plants treated with clothianidin, compared to control and thiamethoxam-treated plants that were also water-stressed and did not have an increase in BGM.

Similar to water-stress, BGM herbivory had a major role in the alteration of plant defenses. As previously observed in tomato and corn plants subjected to spider mite herbivory [7], the presence of BGM increased the concentration of POD and TI. Contrary to TSSM, which was found to trigger the expression of genes codifying for CHI [7], BGM did not appear to promote CHI concentrations. Interestingly, BGM herbivory also increased the total soluble protein concentration in plants when combined with water-stress and neonicotinoids respectively. On one side, water-stressed plants had more proteins than plants provided with optimal irrigation when BGM were present. On the other side, more proteins were found in plants treated with either clothianidin or thiamethoxam than in control plants when subjected to BGM herbivory. The effect of water-stress, which increases the concentration of amino acids in desiccating leaves due to enhanced degradation of ammonia [30], may be one contributing factor leading to the effect of spider mite herbivory, which is known to stimulate the accumulation of amino acids in neighboring cells, following increased osmotic tension and increased sink-demands by the spider mites [55,56]. The greater availability of amino-acids is one possibility that promoted the synthesis of stress proteins, which is commonly triggered in water-stressed plants, as observed in water-stressed corn seedlings [30]. Moreover, spider mites cause cell dehydration by sucking the cell content [57] which, in turn, can lead to additional synthesis of stress proteins. Spider mite feeding is also thought to induce the assimilation of nitrogen into organic nitrogen compounds such as amino acids and peptides, as a compensating mechanism that undamaged mesophyll cells have adapted for the loss of nutrients in damaged neighboring cells [57]. Clothianidin and thiamethoxam, which are N-nitroguanidines, may be factors that exacerbated this process of assimilation by increasing the availability of inorganic nitrogen after the insecticides are degraded in plant tissues.

Some of the variation in plant defenses that we observed between field seasons, can be attributed to soil type and environmental conditions, which can influence the magnitude of the effect of the water-stress severity and duration, as well as plant growth rate [58,59]. In the field specifically, although irrigation treatments were maintained, we recorded more than double the precipitation in 2015 (32.5 mm) than 2013 (14.5 mm). In addition, in 2013 and 2015, relative humidity was 49±1.7% and 54±1.4%, and air temperature was 23±0.4°C and 20±0.3°C, respectively. In a previous study, for example, Gouinguené and Turlings [58] found that differences in soil moisture and air temperature altered the emission of pest induced volatiles in young corn plants. Moreover, higher temperatures are known to interfere with the synthesis of proteins normally produced under non-stressful temperature conditions in favor of novel heat shock proteins [59].

In addition to environmental factors, plant development stage may contribute to the variation in plant defenses. Plants in the field had a lower number of leaves and were shorter than plants in the greenhouse at week 7 after germination, which may have caused a different biosynthesis of plant defenses despite the equal plant age. Commonly more defense compounds are found in younger leaves than in older ones [58–60]. For example, a higher concentration of hydroxamic acids, which play an important role in resistance to western corn rootworm larvae, are commonly found in younger corn plants compared to older ones [61]. Other pathogenesis related proteins, such as glucanase and chitinase, are not detectable in young tobacco leaves, but accumulate with leaf age [62]. In our case, we observed an overall greater concentration of plant defense proteins on greenhouse plants than on equally old plants grown in the field.

When we looked at plant responses, we observed that water-stress decreased plant height, leaf area and leaf number, as well as cob dry weight, which commonly occurs in corn growing under drought conditions [63]. Clothianidin and thiamethoxam did not affect these plant responses, contrary to previous studies where increased leaf area and yield were measured in neonicotinoid-treated plants [64,65]. Similar to water-stress, BGM feeding had an additional major impact on plant responses. In the presence of BGM, plants had lower leaf area, leaf number, and yield, which is similar to what was previously described [66,67]. Surprisingly, BGM feeding resulted in increased leaf temperature, regardless of the irrigation level.

Overall, our study shows that the combination of plant water-stress and neonicotinoids leads to BGM secondary outbreaks in corn, however, the direct interaction may be an additive effect of two plant physiological pathways rather than trade-offs in any one alone. Water-stress appeared to affect some of the key plant defense responses, while neonicotinoids appeared to increase nitrogen concentrations in plants. We conclude that the combination of the two abiotic factors may play a major role in the development of BGM populations in corn through two independent plant pathways.

## Conclusions

Neonicotinoids have come under scrutiny for several unintended consequences on non-target organisms such as pollinators, predators, and now the outbreak of spider mites. These effects, however, can be exacerbated by environmental factors such as drought. As observed in our study, spider mite density in corn was elevated by the combination of two abiotic factors, water-stress and neonicotinoids, which independently caused alterations in plant defense responses and total nitrogen concentrations. However, not all neonicotinoids act in the same way, as was seen with thiamethoxam use and inconsistent BGM outbreaks, compared to clothianidin use. Therefore, understanding the nuances of different neonicotinoid active ingredients is key to selecting insecticide active ingredients have little or no effect on spider mites and that are less harmful to non-target arthropods.

There are a number of ecologically-based pest management strategies for spider mite suppression, including reducing water-stress through proper irrigation, disruption of spider mites in plants with overhead irrigation, and the judicious use of miticides. Given the limitations on water resources, however, the development of commercially available drought-resistant plant hybrids may alleviate spider mite outbreaks in drought-stress conditions. Overall, our new understanding of the interactions between abiotic factors, namely water-stress and neonicotinoid use, on the development of spider mite infestations in corn, allows crop managers to anticipate and predict pest outbreaks and combine this with available pest management strategies.

## Supporting information

S1 Table ANOVA table - Total protein concentration (Field experiment 2). (DOCX)

S2 Table ANOVA table - POD (Field experiment 2). (DOCX)

S3 Table ANOVA table - PPO (Field experiment 2). (DOCX)

S4 Table ANOVA table - CHI (Field experiment 2). (DOCX)

S5 Table ANOVA table - TI (Field experiment 2). (DOCX)

S6 Table ANOVA table - Total protein concentration (Field experiment 3). (DOCX)

S7 Table ANOVA table - POD (Field experiment 3). (DOCX)

S8 Table ANOVA table - PPO (Field experiment 3). (DOCX)

S9 Table ANOVA table - CHI (Field experiment 3). (DOCX)

S10 Table ANOVA table - TI (Field experiment 3). (DOCX)

## References

[1] Douglas MR, Tooker JF (2015) Large-scale deployment of seed treatments has driven rapid increase in use of neonicotinoid insecticides and preemptive pest management in U.S. field crops. Environ. Sci. Technol. Lett. 49(8): 5088–5097. 10.1021/es506141g 25793443

[2] Elbert A, Haas M, Springer B, Thielert W, Nauen R (2008) Applied aspects of neonicotinoid uses in crop protection. Pest. Manag. Sci. 64: 1099–1105. doi: 10.1002/ps.1616 18561166 10.1002/ps.1616

[3] Moser SE, Obrycki JJ (2009) Non-target effects of neonicotinoid seed treatments; mortality of coccinellid larvae related to zoophytophagy. Biol. Control 3:487–92.

[4] Laurino D, Porporato M, Patetta A, Manino A (2011). Toxicity of neonicotinoid insecticides to honey bees: laboratory tests. Bull. Insectology 64(1): 107–113.

[5] Blacquière T, Smagghe G, Cornelis A, van Gestel M, Mommaerts V (2012) Neonicotinoids in bees: a review on concentrations, side-effects and risk assessment. Ecotoxicology 21(4): 973–92. doi: 10.1007/s10646-012-0863-x 22350105 10.1007/s10646-012-0863-x PMC3338325

[6] Szczepaniec A, Creary SF, Laskowski KL, Nyrop JP, Raupp MJ (2011) Neonicotinoid insecticide imidacloprid causes outbreaks of spider mites on elm trees in urban landscapes. PLoS ONE 6(5): e20018 doi: 10.1371/journal.pone.0020018 21655275 10.1371/journal.pone.0020018 PMC3104998

[7] Szczepaniec A, Raupp MJ, Parker RD, Kerns D, Eubanks MD (2013) Neonicotinoid insecticides alter induced defenses and increase susceptibility to spider mites in distantly related crop plants. PLoS ONE 8(5): e62620 doi: 10.1371/journal.pone.0062620 23658754 10.1371/journal.pone.0062620 PMC3643937

[8] Szczepaniec A, Raupp BB, Raupp MJ (2013) Effects of dinotefuran and imidacloprid on target and non-target arthropods on american elm. Arboric. Urban For. 39: 231–235.

[9] Szczepaniec A, Raupp M (2013) Direct and indirect effects of imidacloprid on fecundity and abundance of *Eurytetranychus buxi* (Acari: Tetranychidae) on boxwoods. Exp. Appl. Acarol. 59: 307–318. doi: 10.1007/s10493-012-9614-1 23007227 10.1007/s10493-012-9614-1

[10] Barati R, Hejazi MJ (2015) Reproductive parameters of *Tetranychus urticae* (Acari: Tetranychidae) affected by neonicotinoid insecticides. Exp. Appl. Acarol. 66(4): 481–489. doi: 10.1007/s10493-015-9910-7 25912952 10.1007/s10493-015-9910-7

[11] Smith JF, Catchot FR, Musser JG (2013) Effects of Aldicarb and Neonicotinoid Seed Treatments on Twospotted Spider Mite on Cotton. J Econ Entomol 106(2): 807–815. 23786068 10.1603/ec10125

[12] Poletti M, Maia AHN, Omoto C (2007) Toxicity of neonicotinoid insecticides to *Neoseiulus californicus* and *Phytoseiulus macropilis* (Acari: Phytoseiidae) and their impact on functional response to *Tetranychus urticae* (Acari: Tetranychidae). Biol. Control 40: 30–36.

[13] James DG (2003) Toxicity of imidacloprid to *Galendromus occidentalis*, *Neoseiulus fallacis* and *Amblyseius andersoni* (Acari: Phytoseiidae) from hops in Washington State, USA. Exp. App. Acarol. 31(3-4): 275–281. 10.1023/b:appa.0000010383.33351.2f 14974692

[14] Ullah MS, Hanawa M, Gotoh T (2016) Pesticide-mediated displacement of a phytoseiid predator, *Neoseiulus womersleyi*, by another phytoseiid predator, *N*. *californicus* (Acari: Phytoseiidae). Exp. Appl. Acarol. 69(4): 453–464. doi: 10.1007/s10493-016-0053-2 27207574 10.1007/s10493-016-0053-2

[15] Elzen GW (2001) Lethal and sublethal effects of insecticide residues on *Orius insidiosus* (Hemiptera: Anthocoridae) and *Geocoris punctipes* (Hemiptera: Lygaeidae). J. Econ. Entomol. 94: 55–59. 11233133 10.1603/0022-0493-94.1.55

[16] Stavrinides KM, Daane BD, Lampinen NJM (2010) Plant water stress, leaf temperature, and spider mite (Acari: Tetranychidae) outbreaks in California vineyards. Environ. Entomol. 39 (4): 1232–1241. doi: 10.1603/EN09288 22127173 10.1603/EN09288

[17] Ximénez-Embún MG, Castañera P, Ortego F (2017) Drought stress in tomato increases the performance of adapted and non-adapted strains of *Tetranychus urticae*. J. Insect Physiol. 96: 73–81. doi: 10.1016/j.jinsphys.2016.10.015 27789296 10.1016/j.jinsphys.2016.10.015

[18] Ximénez-Embún MG, Ortego F, Castañera P (2016) Drought-Stressed Tomato Plants Trigger Bottom–Up Effects on the Invasive *Tetranychus evansi*. PLoS ONE 11(1): e0145275 https://doi.org/10.1371/journal.pone.0145275 26735490 10.1371/journal.pone.0145275 PMC4703393

[19] English-Loeb GM (1990) Plant drought- stress and outbreaks of spider mites: a field test. Ecol. 71: 1401–1411.

[20] Chandler LD, Archer TL, Ward CR, Lyle WM (1979) Influences of irrigation practices on spider mite densities on field corn. Environ. Entomol. 8: 196–201.

[21] Price JF, Harbaugh BK, Stanley CD (1982) Response of mites and leafminers to trickle irrigation rates in spray chrysantemum production. HortScience 17: 895–896.

[22] Gutbrodt B, Mody K, Dorn S (2011) Drought changes plant chemistry and causes contrasting responses in lepidopteran herbivores. Oikos 120: 1732–1740.

[23] Rhoades DF, Herbivore population dynamics and plant chemistry In: Denno RF, McClure MS, editors. Variable plants and herbivores in natural and managed systems. New York: Academic Press; 1983 pp. 155–220.

[24] Sánchez-Hernández C, Martínez-Gallardo N, Guerrero-Rangel A, Valdés-Rodríguez S, Délano-Frier J (2004) Trypsin and α-amylase inhibitors are differentially induced in leaves of amaranth (*Amaranthus hypochondriacus*) in response to biotic and abiotic stress. Physiol. Plant 122: 254–264.

[25] Yu L-X, Djebrouni M, Chamberland H, Lafontaine JG, Tabaeizadeh Z (1998) Chitinase: differential induction of gene expression and enzyme activity by drought-stress in the wild (*Lycopersicon chilense* Dun.) and cultivated (*L*. *esculentum* Mill.) tomatoes. J. Plant Physiol. 153: 745–753.

[26] Fazeli F, Ghorbanli M, Niknam V (2007) Effect of drought on biomass, protein content, lipid peroxidation and antioxidant enzymes in two sesame cultivars. Biol. Plant 51: 98–103.

[27] Jaleel CA, Manivannan P, Sankar B, Kishorekumar A, Gopi R, Somasundaram R, et al (2007) Induction of drought-stress tolerance by ketoconazole in *Catharanthus roseus* is mediated by enhanced antioxidant potentials and secondary metabolite accumulation. Colloids Surf. B. Biointerfaces 60: 201–206. doi: 10.1016/j.colsurfb.2007.06.010 17643970 10.1016/j.colsurfb.2007.06.010

[28] White TCR (1969) An index to measure weather-induced stress of trees associated with outbreaks of psyllids in Australia. Ecol. 50: 905–909.

[29] Brodbeck B, Strong D, Amino acid nutrition of herbivorous insects and stress to host plants In: Barbosa P, Schultz JC, editors. Insect Outbreaks. San Diego: Academic Press, Inc 1987 pp. 347–364.

[30] Mohammadkhani N, Heidari R (2008) Effects of drought stress on soluble proteins in two maize varieties. İki Mısır Varyetesinin Çözücü Proteinleri Üzerine Kuraklık Stresinin Etkisi 32: 23–30.

[31] Mattson WJ, Haack RA (1987) The role of drought in outbreaks of plant-eating insects. Biosci. 37: 110–118.

[32] Logan JA, Congdon BD, Allredge JK (1983) Ecology and control of spider mites on corn in northeastern Colorado. Colorado Agri. Exp. Station Bulletin 585S: 1–41.

[33] Croft BA, Baan HEVD (1988) Ecological and genetic factors influencing evolution of pesticide resistance in Tetranychid and Phytoseiid mites. Exp. Appl. Acarol. 4: 277–300.

[34] Khajehali J, Van Nieuwenhuyse P, Demaeght P, Tirry L, Van Leeuwen T (2011) Acaricide resistance and resistance mechanisms in *Tetranychus urticae* populations from rose greenhouses in the Netherlands. Pest Manag. Sci. 67: 1424–1433. doi: 10.1002/ps.2191 21548003 10.1002/ps.2191

[35] National oceanic and atmospheric administration (2014) U.S. seasonal drought outlook. Accessed: http://www.cpc.ncep.noaa.gov/.

[36] Blasi ÉAR, Buffon G, da Silva RZ, Stein C, Dametto A, Ferla NJ, et al (2014) Alterations in rice, corn and wheat plants infested by phytophagous mite. Int. J. Acarol. 41: 10–18.

[37] Allen RG, Pereira LS, Raes D, Smith M (1998) Crop evapotranspiration - Guidelines for computing crop water requirements. FAO Irrigation and Drainage 56: 1–326.

[38] Reynolds S G (1970) The gravimetric method of soil moisture determination. J Hydrol 11: 258–273.

[39] Wrensch D, Young SSY (1975) Effects of quality of resource and fertilization status on some fitness traits in the twospotted spider mite, *Tetranychus urticae* Koch. Oecologia 18: 259–267. doi: 10.1007/BF00345850 28308917 10.1007/BF00345850

[40] Gerhards M., Rock G., Schlerf M., Udelhoven T. (2016) Water stress detection in potato using leaf temperature, emissivity, and reflectance. Intern. J. App. Earth Obs. Geoinf. 53:27–39.

[41] Stiefel VL (1992) Leaf temperature affects resistance to the Banks grass mite (Acari, Tetranychidae) on drought-resistant grain-sorghum. J. Econ. Entomol. 85: 2170–2184.

[42] Walker WR., and Skogerboe GV (1987). Surface irrigation: theory and practice Englewood Cliffs, NJ, USA: Prentice-Hall Inc xiii, 386p.

[43] Mondal M, and Ara N (2006) Biology and fecundity of the twospotted spider mite, *Tetranychus urticae* Koch. (Acari: Tetranychidae) under laboratory conditions. J. Life Earth Sci. 1(2): 43–47.

[44] Barto EK, Cipollini D. (2005) Testing the optimal defense theory and the growth-differentiation balance hypothesis in *Arabidopsis thaliana*. Oecologia 146: 169 doi: 10.1007/s00442-005-0207-0 16096848 10.1007/s00442-005-0207-0

[45] Bradford MM (1976) A rapid and sensitive method for the quantitation of microgram quantities of protein utilizing the principle of protein dye-binding. Anal. Biochem. 72: 248–254. 942051 10.1016/0003-2697(76)90527-3

[46] Moran PJ, Cipollini DF (1999) Effect of wind-induced mechanical stress on soluble peroxidase activity and resistance to pests in cucumber. J. Phytopathol. 147: 313–316.

[47] dal Soglio FK, Bertagnolli BL, Sinclair JB, Yu GY, Eastburn DM (1998) Production of chitinolytic enzymes and endoglucanase in the soybean rhizosphere in the presence of trichoderma harzianumand (*Rhizoctonia solani*). Biol. Control 12: 111–117.

[48] Cipollini D, Bergelson J (2000) Environmental and developmental regulation of trypsin inhibitor activity in Brassica napus. J. Chemical Ecol. 26: 1411–1422.

[49] Nauen R, Ebbinghaus-Kintscher U, Salgado VL, Kaussmann M (2003) Thiamethoxam is a neonicotinoid precursor converted to clothianidin in insects and plants. Pestic. Biochem. Physiol. 76: 55–69.

[50] Ferro D N, and Chapman R B (1979) Effects of different constant humidities and temperatures on twospotted spider mite egg hatch. Environ. Entomol. 8:701–705.

[51] Perring T M, Holtzer T O, Toole J L, Norman J M, Myers G L (1984) Influences of temperature and humidity on pre-adult development of the Banks grass mite (Acari: Tetranychidae). Environ. Entomol. 13: 338–343.

[52] Gershenzon J (1984) Changes in the levels of plant secondary metabolites under water and nutrient stress. Recent Adv. Phytochem. 18: 273–320.

[53] Ford KA, Casida JE, Chandran D, Gulevich AG, Okrent RA, Durkin KA, et al (2010) Neonicotinoid insecticides induce salicylate-associated plant defense responses. Proc. Natl. Acad. Sci. U.S.A. 107: 17527–17532. doi: 10.1073/pnas.1013020107 20876120 10.1073/pnas.1013020107 PMC2955088

[54] Stout MJ, Workman J, Duffey SS (1994) Differential induction of tomato foliar proteins by arthropod herbivores. J. Chem. Ecol. 20: 2575–2594. doi: 10.1007/BF02036193 24241833 10.1007/BF02036193

[55] Kolodziej A, Kropezyńska D, Poskuta J (1975) The effect of twospotted spider mite (*Tetranychus urticae* Koch) injury on carbon metabolism in *Chrysanthemum morifolium* L. In:Proc. 7th Int. Congr. Plant Protect., Moscow, pp. 217-229.

[56] Borichenko N and Manolov P (1982) Changes in some physiological indicators and biochemical processes of apple leaves infested in various extents by *Panonychus ulmi* Koch. Horti. Viticultural Sci. 19: 44–50.

[57] Tomczyk A and Kropczyńska D. Effects on the host plants In: Helle W. Sabelis MW editors. Spider mites, their biology, natural enemies and control. Amsterdam: Elsevier, 1985, vol. 1A pp. 317–327.

[58] Gouinguené SP, Turlings TCJ (2012) The effects of abiotic factors on induced volatile emissions in corn plants. Plant Physiol. 129(3):1296–1307. 10.1104/pp.001941 PMC166523 12114583

[59] Bray EA, Bailey-Serres J., Weretilnyk E (2002). Responses to abiotic stresses In Gruissem W, Buchannan BB., Jones RL editors. Biochemistry and molecular biology of plants. Rockville: Curr. Topics Plant Physiol. 1158-1203.

[60] Krischik VA, Denno RF (1983) Individual, population,and geographic patterns in plant defense In: Denno RF, McClure MS editors. Variable plants and herbivores in natural and managed systems. San Diego: Academic Press, Inc 1983 pp. 463–512.

[61] Cambier V, Hance T, De Hoffmann E (2000) Variation of DIMBOA and related compounds content in relation to the age and plant organ in maize. Phytochemistry 53: 223–229. 10680175 10.1016/s0031-9422(99)00498-7

[62] Van Loon LC, Rep M, Pieterse CMJ (2006) Significance of inducible defense-related proteins in infected plants. Annu. Rev. Phytopathol. 44: 135–162. doi: 10.1146/annurev.phyto.44.070505.143425 16602946 10.1146/annurev.phyto.44.070505.143425

[63] Ҫakir R (2004) Effect of water stress at different development stages on vegetative and reproductive growth of corn. Field Crops Res. 8(1): 1–16.

[64] Gonias ED, Oosterhuis DM (2008). Physiologic response of cotton to the insecticide imidacloprid under high-temperature stress. J. Plant Growth Regul. 27(1): 77–82.

[65] Gonias ED, Oosterhuis DM, Bibi DC (2006) How the insecticide Trimax improves growth and yield in cotton. Proceedings of the Beltwide Cotton Conference, San Antonio, TX. National Cotton Council of America, Memphis, TN.

[66] Avery DJ and Briggs JB (1968) Damage to leaves caused by fruit tree red spider mite, *Panonychus ulmi* (Koch). J. Hortic. Sci. 43: 463–473.

[67] Summers FM and Stocking CR (1972) Some immediate effects of almond feeding by *Bryobra rubrioculus* (Scheuten). Acarologia 14:170–178.

