# Supplementary material for: Combinations of plant water-stress and neonicotinoids can lead to secondary outbreaks of Banks grass mite (Oligonychus pratensis Banks)
Source: PLoS One. 2018 Feb 28;13(2):e0191536. doi: 10.1371/journal.pone.0191536 (PMC5830035; doi:10.1371/journal.pone.0191536)
Supplement: S1 Table — (DOCX) [file pone.0191536.s001.docx]

**Table S1. ANOVA table - Total protein concentration (Field experiment 2).**

| **Type III Tests of Fixed Effects** | | | | |
| --- | --- | --- | --- | --- |
| **Effect** | **Num DF** | **Den DF** | **F Value** | **Pr > F** |
| **water** | 1 | 93 | 2.90 | 0.0921 |
| **pesticide** | 1 | 93 | 0.91 | 0.3433 |
| **pesticide*water** | 1 | 93 | 2.10 | 0.1509 |
| **herbivory** | 1 | 93 | 8.61 | 0.0042 |
| **water*herbivory** | 1 | 93 | 5.13 | 0.0258 |
| **pesticide*herbivory** | 1 | 93 | 0.25 | 0.6177 |
| **pestic*water*herbivo** | 1 | 93 | 0.00 | 0.9821 |
| **time** | 2 | 93 | 147.36 | <.0001 |
| **water*time** | 2 | 93 | 0.66 | 0.5184 |
| **pesticide*time** | 2 | 93 | 0.05 | 0.9515 |
| **pesticide*water*time** | 2 | 93 | 1.11 | 0.3354 |
| **herbivory*time** | 2 | 93 | 1.86 | 0.1608 |
| **water*herbivory*time** | 2 | 93 | 1.25 | 0.2905 |
| **pestici*herbivo*time** | 2 | 93 | 3.79 | 0.0262 |
| **pest*wate*herbi*time** | 2 | 93 | 1.65 | 0.1969 |
