# Supplementary material for: Combinations of plant water-stress and neonicotinoids can lead to secondary outbreaks of Banks grass mite (Oligonychus pratensis Banks)
Source: PLoS One. 2018 Feb 28;13(2):e0191536. doi: 10.1371/journal.pone.0191536 (PMC5830035; doi:10.1371/journal.pone.0191536)
Supplement: S2 Table — (DOCX) [file pone.0191536.s002.docx]

**S2 Table. ANOVA table - POD (Field experiment 2)**

| **Type III Tests of Fixed Effects** | | | | |
| --- | --- | --- | --- | --- |
| **Effect** | **Num DF** | **Den DF** | **F Value** | **Pr > F** |
| **water** | 1 | 90 | 11.11 | 0.0012 |
| **pesticide** | 1 | 90 | 1.70 | 0.1958 |
| **pesticide*water** | 1 | 90 | 0.51 | 0.4759 |
| **herbivory** | 1 | 90 | 16.11 | 0.0001 |
| **water*herbivory** | 1 | 90 | 0.80 | 0.3720 |
| **pesticide*herbivory** | 1 | 90 | 0.00 | 0.9443 |
| **pestic*water*herbivo** | 1 | 90 | 2.20 | 0.1417 |
| **time** | 2 | 90 | 29.08 | <.0001 |
| **water*time** | 2 | 90 | 2.78 | 0.0672 |
| **pesticide*time** | 2 | 90 | 0.73 | 0.4860 |
| **pesticide*water*time** | 2 | 90 | 0.15 | 0.8627 |
| **herbivory*time** | 2 | 90 | 1.17 | 0.3136 |
| **water*herbivory*time** | 2 | 90 | 2.49 | 0.0884 |
| **pestici*herbivo*time** | 2 | 90 | 0.21 | 0.8137 |
| **pest*wate*herbi*time** | 2 | 90 | 7.89 | 0.0007 |
