# Supplementary material for: Combinations of plant water-stress and neonicotinoids can lead to secondary outbreaks of Banks grass mite (Oligonychus pratensis Banks)
Source: PLoS One. 2018 Feb 28;13(2):e0191536. doi: 10.1371/journal.pone.0191536 (PMC5830035; doi:10.1371/journal.pone.0191536)
Supplement: S3 Table — (DOCX) [file pone.0191536.s003.docx]

**S3 Table. ANOVA table - PPO (Field experiment 2)**

| **Type III Tests of Fixed Effects** | | | | |
| --- | --- | --- | --- | --- |
| **Effect** | **Num DF** | **Den DF** | **F Value** | **Pr > F** |
| **water** | 1 | 93 | 1.25 | 0.3676 |
| **pesticide** | 1 | 93 | 4.48 | 0.9903 |
| **pesticide*water** | 1 | 93 | 8.41 | 0.1818 |
| **herbivory** | 1 | 93 | 22.72 | 0.7641 |
| **water*herbivory** | 1 | 93 | 30.04 | 0.3408 |
| **pesticide*herbivory** | 1 | 93 | 2.57 | 0.4537 |
| **pestic*water*herbivo** | 1 | 93 | 1.47 | 0.5284 |
| **time** | 2 | 93 | 9.69 | 0.0002 |
| **water*time** | 2 | 93 | 0.99 | 0.8200 |
| **pesticide*time** | 2 | 93 | 4.68 | 0.2881 |
| **pesticide*water*time** | 2 | 93 | 0.71 | 0.3924 |
| **herbivory*time** | 2 | 93 | 36.04 | <.0001 |
| **water*herbivory*time** | 2 | 93 | 0.78 | 0.6641 |
| **pestici*herbivo*time** | 2 | 93 | 1.11 | 0.4334 |
| **pest*wate*herbi*time** | 2 | 93 | 3.16 | 0.4507 |
