# Supplementary material for: Combinations of plant water-stress and neonicotinoids can lead to secondary outbreaks of Banks grass mite (Oligonychus pratensis Banks)
Source: PLoS One. 2018 Feb 28;13(2):e0191536. doi: 10.1371/journal.pone.0191536 (PMC5830035; doi:10.1371/journal.pone.0191536)
Supplement: S4 Table — (DOCX) [file pone.0191536.s004.docx]

**S4 Table. ANOVA table - CHI (Field experiment 2)**

| **Type III Tests of Fixed Effects** | | | | |
| --- | --- | --- | --- | --- |
| **Effect** | **Num DF** | **Den DF** | **F Value** | **Pr > F** |
| **water** | 1 | 93 | 1.25 | 0.2672 |
| **pesticide** | 1 | 93 | 0.48 | 0.4903 |
| **pesticide*water** | 1 | 93 | 1.81 | 0.1818 |
| **herbivory** | 1 | 93 | 24.74 | <.0001 |
| **water*herbivory** | 1 | 93 | 3.30 | 0.0725 |
| **pesticide*herbivory** | 1 | 93 | 0.57 | 0.4537 |
| **pestic*water*herbivo** | 1 | 93 | 1.47 | 0.2284 |
| **time** | 2 | 93 | 9.69 | 0.0002 |
| **water*time** | 2 | 93 | 0.19 | 0.8300 |
| **pesticide*time** | 2 | 93 | 1.61 | 0.2061 |
| **pesticide*water*time** | 2 | 93 | 0.71 | 0.4954 |
| **herbivory*time** | 2 | 93 | 0.06 | 0.9456 |
| **water*herbivory*time** | 2 | 93 | 0.58 | 0.5647 |
| **pestici*herbivo*time** | 2 | 93 | 1.11 | 0.3334 |
| **pest*wate*herbi*time** | 2 | 93 | 0.06 | 0.9450 |
