# Supplementary material for: Combinations of plant water-stress and neonicotinoids can lead to secondary outbreaks of Banks grass mite (Oligonychus pratensis Banks)
Source: PLoS One. 2018 Feb 28;13(2):e0191536. doi: 10.1371/journal.pone.0191536 (PMC5830035; doi:10.1371/journal.pone.0191536)
Supplement: S5 Table — (DOCX) [file pone.0191536.s005.docx]

**S5 Table. ANOVA table - TI (Field experiment 2)**

| **Type III Tests of Fixed Effects** | | | | |
| --- | --- | --- | --- | --- |
| **Effect** | **Num DF** | **Den DF** | **F Value** | **Pr > F** |
| **water** | 1 | 31 | 0.89 | 0.3536 |
| **pesticide** | 1 | 31 | 2.89 | 0.0994 |
| **pesticide*water** | 1 | 31 | 1.92 | 0.1754 |
| **herbivory** | 1 | 31 | 0.00 | 0.9944 |
| **water*herbivory** | 1 | 31 | 6.49 | 0.0160 |
| **pesticide*herbivory** | 1 | 31 | 2.32 | 0.1375 |
| **pestic*water*herbivo** | 1 | 31 | 4.24 | 0.0481 |
| **time** | 2 | 62 | 85.71 | <.0001 |
| **water*time** | 2 | 62 | 1.56 | 0.2193 |
| **pesticide*time** | 2 | 62 | 0.20 | 0.8177 |
| **pesticide*water*time** | 2 | 62 | 0.62 | 0.5395 |
| **herbivory*time** | 2 | 62 | 5.71 | 0.0053 |
| **water*herbivory*time** | 2 | 62 | 0.71 | 0.4934 |
| **pestici*herbivo*time** | 2 | 62 | 1.17 | 0.3177 |
| **pest*wate*herbi*time** | 2 | 62 | 1.27 | 0.2873 |
