# Supplementary material for: Combinations of plant water-stress and neonicotinoids can lead to secondary outbreaks of Banks grass mite (Oligonychus pratensis Banks)
Source: PLoS One. 2018 Feb 28;13(2):e0191536. doi: 10.1371/journal.pone.0191536 (PMC5830035; doi:10.1371/journal.pone.0191536)
Supplement: S6 Table — (DOCX) [file pone.0191536.s006.docx]

**S6 Table. ANOVA table - Total protein concentration (Field experiment 3)**

| **Type III Tests of Fixed Effects** | | | | |
| --- | --- | --- | --- | --- |
| **Effect** | **Num DF** | **Den DF** | **F Value** | **Pr > F** |
| **water** | 1 | 72 | 2.42 | 0.1242 |
| **pesticide** | 2 | 72 | 0.02 | 0.9819 |
| **pesticide*water** | 2 | 72 | 0.96 | 0.3894 |
| **herbivory** | 1 | 72 | 0.05 | 0.8178 |
| **water*herbivory** | 1 | 72 | 0.03 | 0.8682 |
| **pesticide*herbivory** | 2 | 72 | 1.52 | 0.2264 |
| **pestic*water*herbivo** | 2 | 72 | 0.31 | 0.7308 |
| **time** | 2 | 72 | 6.06 | 0.0037 |
| **water*time** | 2 | 72 | 3.42 | 0.0382 |
| **pesticide*time** | 4 | 72 | 1.28 | 0.2846 |
| **pesticide*water*time** | 4 | 72 | 3.24 | 0.0168 |
| **herbivory*time** | 2 | 72 | 1.96 | 0.1487 |
| **water*herbivory*time** | 2 | 72 | 4.66 | 0.0125 |
| **pestici*herbivo*time** | 4 | 72 | 1.90 | 0.1199 |
| **pest*wate*herbi*time** | 4 | 72 | 0.29 | 0.8840 |
