# Supplementary material for: Combinations of plant water-stress and neonicotinoids can lead to secondary outbreaks of Banks grass mite (Oligonychus pratensis Banks)
Source: PLoS One. 2018 Feb 28;13(2):e0191536. doi: 10.1371/journal.pone.0191536 (PMC5830035; doi:10.1371/journal.pone.0191536)
Supplement: S7 Table — (DOCX) [file pone.0191536.s007.docx]

**S7 Table. ANOVA table - POD (Field experiment 3)**

| **Type III Tests of Fixed Effects** | | | | |
| --- | --- | --- | --- | --- |
| **Effect** | **Num DF** | **Den DF** | **F Value** | **Pr > F** |
| **water** | 1 | 68 | 41.21 | <.0001 |
| **pesticide** | 2 | 68 | 1.35 | 0.2668 |
| **pesticide*water** | 2 | 68 | 0.88 | 0.4190 |
| **herbivory** | 1 | 68 | 0.06 | 0.8139 |
| **water*herbivory** | 1 | 68 | 0.02 | 0.8832 |
| **pesticide*herbivory** | 2 | 68 | 4.03 | 0.0223 |
| **pestic*water*herbivo** | 2 | 68 | 1.52 | 0.2271 |
| **time** | 2 | 68 | 38.25 | <.0001 |
| **water*time** | 2 | 68 | 14.28 | <.0001 |
| **pesticide*time** | 4 | 68 | 2.12 | 0.0873 |
| **pesticide*water*time** | 4 | 68 | 5.06 | 0.0012 |
| **herbivory*time** | 2 | 68 | 10.56 | 0.0001 |
| **water*herbivory*time** | 2 | 68 | 4.00 | 0.0228 |
| **pestici*herbivo*time** | 4 | 68 | 6.57 | 0.0002 |
| **pest*wate*herbi*time** | 4 | 68 | 1.69 | 0.1617 |
