# Supplementary material for: Combinations of plant water-stress and neonicotinoids can lead to secondary outbreaks of Banks grass mite (Oligonychus pratensis Banks)
Source: PLoS One. 2018 Feb 28;13(2):e0191536. doi: 10.1371/journal.pone.0191536 (PMC5830035; doi:10.1371/journal.pone.0191536)
Supplement: S8 Table — (DOCX) [file pone.0191536.s008.docx]

**S8 Table. ANOVA table - PPO (Field experiment 3)**

| **Type III Tests of Fixed Effects** | | | | |
| --- | --- | --- | --- | --- |
| **Effect** | **Num DF** | **Den DF** | **F Value** | **Pr > F** |
| **water** | 1 | 72 | 10.79 | 0.0016 |
| **pesticide** | 2 | 72 | 0.10 | 0.9051 |
| **pesticide*water** | 2 | 72 | 0.90 | 0.4128 |
| **herbivory** | 1 | 72 | 7.79 | 0.0067 |
| **water*herbivory** | 1 | 72 | 0.59 | 0.4451 |
| **pesticide*herbivory** | 2 | 72 | 4.90 | 0.0101 |
| **pestic*water*herbivo** | 2 | 72 | 2.16 | 0.1232 |
| **time** | 2 | 72 | 267.29 | <.0001 |
| **water*time** | 2 | 72 | 18.12 | <.0001 |
| **pesticide*time** | 4 | 72 | 0.13 | 0.9722 |
| **pesticide*water*time** | 4 | 72 | 0.11 | 0.9797 |
| **herbivory*time** | 2 | 72 | 0.41 | 0.6685 |
| **water*herbivory*time** | 2 | 72 | 1.01 | 0.3697 |
| **pestici*herbivo*time** | 4 | 72 | 3.93 | 0.0061 |
| **pest*wate*herbi*time** | 4 | 72 | 4.89 | 0.0015 |
