# Supplementary material for: Combinations of plant water-stress and neonicotinoids can lead to secondary outbreaks of Banks grass mite (Oligonychus pratensis Banks)
Source: PLoS One. 2018 Feb 28;13(2):e0191536. doi: 10.1371/journal.pone.0191536 (PMC5830035; doi:10.1371/journal.pone.0191536)
Supplement: S9 Table — (DOCX) [file pone.0191536.s009.docx]

**S9 Table. ANOVA table - CHI (Field experiment 3)**

| **Type III Tests of Fixed Effects** | | | | |
| --- | --- | --- | --- | --- |
| **Effect** | **Num DF** | **Den DF** | **F Value** | **Pr > F** |
| **water** | 1 | 72 | 0.10 | 0.7564 |
| **pesticide** | 2 | 72 | 0.79 | 0.4588 |
| **pesticide*water** | 2 | 72 | 0.40 | 0.6725 |
| **herbivory** | 1 | 72 | 2.49 | 0.1192 |
| **water*herbivory** | 1 | 72 | 2.59 | 0.1121 |
| **pesticide*herbivory** | 2 | 72 | 0.33 | 0.7224 |
| **pestic*water*herbivo** | 2 | 72 | 0.65 | 0.5235 |
| **time** | 2 | 72 | 1.00 | 0.3723 |
| **water*time** | 2 | 72 | 5.17 | 0.0080 |
| **pesticide*time** | 4 | 72 | 1.38 | 0.2478 |
| **pesticide*water*time** | 4 | 72 | 3.55 | 0.0106 |
| **herbivory*time** | 2 | 72 | 1.49 | 0.2328 |
| **water*herbivory*time** | 2 | 72 | 6.15 | 0.0034 |
| **pestici*herbivo*time** | 4 | 72 | 0.72 | 0.5820 |
| **pest*wate*herbi*time** | 4 | 72 | 0.92 | 0.4574 |
