# Supplementary material for: Combinations of plant water-stress and neonicotinoids can lead to secondary outbreaks of Banks grass mite (Oligonychus pratensis Banks)
Source: PLoS One. 2018 Feb 28;13(2):e0191536. doi: 10.1371/journal.pone.0191536 (PMC5830035; doi:10.1371/journal.pone.0191536)
Supplement: S10 Table — (DOCX) [file pone.0191536.s010.docx]

**S10 Table. ANOVA table - TI (Field experiment 3)**

| **Type III Tests of Fixed Effects** | | | | |
| --- | --- | --- | --- | --- |
| **Effect** | **Num DF** | **Den DF** | **F Value** | **Pr > F** |
| **water** | 1 | 71 | 0.07 | 0.7887 |
| **pesticide** | 2 | 71 | 1.11 | 0.3366 |
| **pesticide*water** | 2 | 71 | 1.18 | 0.3120 |
| **herbivory** | 1 | 71 | 3.90 | 0.0522 |
| **water*herbivory** | 1 | 71 | 2.92 | 0.0920 |
| **pesticide*herbivory** | 2 | 71 | 0.04 | 0.9621 |
| **pestic*water*herbivo** | 2 | 71 | 0.06 | 0.9403 |
| **time** | 2 | 71 | 17.26 | <.0001 |
| **water*time** | 2 | 71 | 3.48 | 0.0363 |
| **pesticide*time** | 4 | 71 | 1.08 | 0.3718 |
| **pesticide*water*time** | 4 | 71 | 3.28 | 0.0159 |
| **herbivory*time** | 2 | 71 | 0.20 | 0.8185 |
| **water*herbivory*time** | 2 | 71 | 14.48 | <.0001 |
| **pestici*herbivo*time** | 4 | 71 | 3.14 | 0.0196 |
| **pest*wate*herbi*time** | 4 | 71 | 0.28 | 0.8924 |
